# Acute physiological outcomes of high-intensity functional training: a scoping review

**DOI:** 10.7717/peerj.14493

**Published:** 2023-01-03

**Authors:** Jacob M. McDougle, Gerald T. Mangine, Jeremy R. Townsend, Adam R. Jajtner, Yuri Feito

**Affiliations:** 1 Exercise Science and Sport Management, Kennesaw State University, Kennesaw, GA, United States; 2 Exercise and Nutrition Science, Lipscomb University, Nashville, TN, United States; 3 Exercise Physiology, Kent State University, Kent, OH, United States; 4 American College of Sports Medicine, Indianapolis, IN, United States

**Keywords:** CrossFit, Exercise intensity, Methodology, Resistance exercise, HIIT

## Abstract

**Background:**

Systematic reviews and meta-analyses related to high-intensity functional training (HIFT) have been conducted. However, due to a restricted pool of available research, these investigations are often limited in scope. As such, a scoping review investigating the present literature surrounding the acute physiological response to HIFT-based exercise was chosen as a more appropriate structured review.

**Methodology:**

A scoping review was conducted following Arksey and O’Malley’s framework. Three large scale databases were searched to reveal any article pertaining to HIFT and related exercise terminology.

**Results:**

A total of 2,241 articles were found during the initial search. Following this, titles, then abstracts, and full-texts were reviewed to determine inclusion eligibility. A total of 60 articles which investigated a combined total of 35 unique HIFT workouts were included within this review.

**Conclusions:**

A variety of physiological parameters and HIFT workouts have been examined. Markers of intensity (*e.g*., blood lactate concentrations, heart rate) have been most consistently assessed across all studies, and these support the idea that HIFT workouts are typically performed at high-intensity. In contrast, the inclusion of most other measures (*e.g*., hormonal, markers of inflammation and damage, energy expenditure, performance) has been inconsistent and has thus, limited the possibility for making generalized conclusions. Differences in study methodologies have further impacted conclusions, as different studies have varied in sample population characteristics, workouts assessed, and time points. Though it may be impossible to comprehensively research all possible HIFT workouts, consistent adoption of population definitions and workload quantification may overcome this challenge and assist with future comparisons.

## Introduction

Over the past 50 years, a variety of high-intensity training programs have emerged to become the leading fitness trends (Thompson, 2021). Though many forms exist, programming is commonly assigned at vigorous to near-maximal training intensities (≥60–90% heart rate reserve, ≥77–96% maximal heart rate) (Liguori, 2021), and may be differentiated by modality, frequency, duration, rest intervals, and variation. Two noteworthy examples are high-intensity interval training (HIIT) and high-intensity functional training (HIFT) because they both encourage high-intensity effort but are characterized by different modality and work-rest parameters. HIIT involves short, high-intensity efforts separated by predefined rest periods using a single modality (*e.g*., cycling or running) (Buchheit & Laursen, 2013; Buchheit & Laursen, 2013; Babraj et al., 2009; Gillen & Gibala, 2014), whereas HIFT variably incorporates numerous modalities (*e.g*., cycling and running, gymnastics and calisthenics, weightlifting, *etc.*) and typically allows trainees to auto-regulate rest so that effort may range from continuous to intermittent (Feito et al., 2018b). HIFT protocols may also specify work and rest intervals at times, and when this occurs, these are referred as multimodal HIIT.

Compared to traditional, moderate-intensity continuous training, HIIT, multimodal HIIT, and HIFT may be more attractive. These programs require significantly less time to elicit the same benefits and have been reported to be more enjoyable (Gillen & Gibala, 2014; Ballesta-Garcia et al., 2019; Brito et al., 2014; Heinrich et al., 2014). However, differentiating the advantages and disadvantages among high-intensity regimens is more difficult because of their flexible programming structure. For instance, a variety of beneficial fitness adaptations have been reported following all three training forms mentioned (Gillen & Gibala, 2014; Feito et al., 2018c; Buckley et al., 2015; Louzada et al., 2020; Schaun et al., 2018), however, neither HIIT nor multimodal HIIT have been directly compared to HIFT. This is most likely because of the inherent differences that exist among these programming strategies. To make fair comparisons, an essential characteristic of one strategy must be sacrificed to equate workloads. Moreover, with nearly infinite possible combinations available for comparisons, numerous studies would be needed to make generalized conclusions and existing HIFT research is relatively incomplete compared to more traditional exercise forms. Thus, it is essential to begin with a clear definition of HIFT.

### Definition of high-intensity functional training

HIFT incorporates a variety of functional, multimodal movements, intended to be performed at a relatively high-intensity to elicit improvements in general physical preparedness (Feito et al., 2018b). In practice, only a portion of this definition may be recognizable in daily workouts, whereas its entirety is more apparent over the course of several weeks to months of training. That is, single workouts might only address a few desired training outcomes in less than a handful of motor patterns. If viewed individually or only in the short-term (*e.g*., 1 week or month), one might conclude that relevant movement patterns and physiological traits are not being equally addressed in training. However, viewed over a longer time interval, a well-designed HIFT program should equally address all relevant motor patterns and physiological traits. Regardless of this, however, three key characteristics may be extracted from this definition that differentiate the HIFT scheme from others. These include the format or structure of workouts, the types or classification of exercises that compose workouts, and the variability in workout stimulus to promote general physical preparedness.

#### Workout structure

Workout structure is a key difference between HIFT and other types of high-intensity exercise programs. Workouts are often organized into one or more circuits of exercises with completion instructions that emphasize density (*i.e*., completing repetitions at the fastest pace possible). One common structure creates a list of exercises with specific prescription (*e.g*., loads, repetitions, durations) and has trainees repeat the list for ‘*as many repetitions as possible*’ (AMRAP) for a stated duration. Another common structure might ask trainees to complete that same list (or repeat it a specific number of times) as fast as possible and record their time to completion (TTC). The CrossFit® benchmark workouts “Cindy” and “Fran” are two of the most common workouts found within existing HIFT research (CrossFit, 2019; Kliszczewicz et al., 2015; Zeitz et al., 2020; Cavedon et al., 2020), and they provide excellent examples of the AMRAP and TTC workout structures, respectively. “Cindy” is a 20-min AMRAP of five pull-ups, 10 push-ups, and 15 air squats (CrossFit, 2019). Like most AMRAP workouts, trainees must complete all assigned repetitions for each exercise in their prescribed order before returning to the first exercise (*i.e*., five pull-ups, then 10 push-ups, and then 15 air squats before returning to pull-ups). At the end of 20 min, trainees record the total number of repetitions completed as their score. “Fran” is also a circuit, and it consists of two exercises (barbell thrusters and pull-ups) that are repeated sequentially over three rounds, where trainees progressively complete less repetitions (*i.e*., 21-15-9 repetitions) for both exercises on each round. However, instead of repeating this prescription for a specified duration, trainees are tasked with completing the 21-15-9-repetition circuit once and are scored by TTC. Other common structures include the ‘*every minute on the minute*’ (EMOM) format and an adapted Tabata-style protocol (Viana et al., 2019). The EMOM structure assigns a specific number of repetitions to an exercise that must be completed within 1 min and repeats this prescription every minute for a pre-defined duration. The EMOM structure is also flexible in that interval durations are not limited to lasting 1 min only. An E2MOM repeats prescription every 2 min on the minute, whereas the prescribed repetitions of “Death by…” workouts increase the repetitions to be completed on each minute. Meanwhile, Tabata-style workouts may be viewed as a subcategory of the AMRAP structure that assign a specific exercise to be completed for ‘*as many repetitions as possible*’ within eight, consecutive 20-s rounds. Rounds are separated by 10-s rest intervals for a total duration of 4 min. While any of these and other structures may be employed, AMRAP’s and TTC’s workouts are the most common HIFT structures (CrossFit, 2022; WODwell, 2022).

#### Exercise selection

Although many definitions exist, ‘functional’ exercises are typically whole-body movements that activate multiple muscle groups through universal motor recruitment patterns mirroring activities of daily living (*e.g*., squatting to sit on a toilet or chair, picking an object off the ground, carrying groceries to and from a vehicle, *etc.*) (Heinrich et al., 2014; Poston et al., 2016). The intention behind emphasizing functional movements is to elicit greater expressions of force and power (CrossFit, 2002), which may better promote developments in neuromuscular function, muscle mass and quality, and strength compared to non-functional exercises (Metter et al., 1997; Heinrich et al., 2021). Over the course of training, HIFT workouts may incorporate a wide array of exercises from multiple modalities that vary in ‘functional’ degree. While an exercise’s degree of function will impact its ability to stimulate adaptations (*i.e*., less functional exercises provide a lesser stimulus) (CrossFit, 2002), variability in functional degree may be purposeful and based on the trainee’s skill in performing certain movement patterns and the specific goals of the workout. For example, the muscle-up exercise requires a powerful, full-body swing (or “kip”) to accelerate the trainee from a hanging position (from a pair of rings or pull-up bar) to a controlled, upright position where their arms are extended, and lower torso/hips are even with the rings or bar. The less complex segments of this exercise (*i.e*., the ‘kip’, hip pull, turnover, *etc.*) reduce range of motion, musculature involved, contraction velocity, relative intensity, and work completed (CrossFit, 2019), and are, thus, less functional. Nevertheless, less complex segments, or similar but less complex motions (*e.g*., pull-ups), may be programmed as learning tools or because the trainee cannot complete repetitions at the workout’s intended pace.

HIFT workouts draw from a wide array of exercises that fall into one of three categories: weightlifting, gymnastics, or monostructural. In addition to its traditional definition (*i.e*., Olympic weightlifting), weightlifting exercises in HIFT refer to any exercise that uses an external load as a means of resistance (CrossFit, 2019). This usually involves variations of typical Olympic weightlifting and power lifts, but also includes exercises that utilize kettlebells, dumbbells, or medicine balls. Gymnastic (and calisthenic) movements are those that utilize the trainee’s body mass as the resistance and, at times, an external object meant to serve as an obstacle. For instance, no additional equipment is needed for a “burpee” and body mass is the primary source of intensity. However, when the task is elevated to a “burpee box jump-over”, the box serves as the obstacle that the trainee may land upon or simply jump over after performing a burpee. Alternatively, pull-ups also require an external object (a hanging pull-up bar) upon which the trainee moves their body. Meanwhile, monostructural exercises refer to exercise that is continuous, repetitive, and cyclical in nature (*e.g*., rowing, running, biking, swimming, or skiing). Any combination of these three categories may appear in a single workout. Some may include only one modality whereas others may draw from two or all three modalities in equal or unequal amounts.

#### General physical preparedness

Several philosophies about progression and periodization exist within the realm of exercise prescription (Haff, 2015a; Haff, 2015b). The main tenet of progression (or progressive overload) is that training must consistently challenge a targeted physiological trait to elicit continued adaptation. HIFT simultaneously allows this to occur constantly and at the trainee’s discretion (Feito et al., 2018b; CrossFit, 2019). The overloading stimulus of a workout is accomplished by its instructions (*i.e*., AMRAP or TTC) but because the trainee may auto-regulate rest intervals and is free to scale intensity, duration, and complexity, the degree of overload may range drastically. The trainee’s discretion also necessarily affects any periodization strategy that might be employed to achieve HIFT’s defining characteristic, to develop general physical preparedness. While individual workouts may be designed to only challenge one or a few targeted physiological traits, the accumulation of several workouts across training is intended to challenge and stimulate simultaneous adaptations across all areas of fitness (*e.g*., cardiorespiratory and circulatory fitness, metabolic function, neuromuscular function and quality, *etc.*) and sport-specific skill (Feito et al., 2018b; CrossFit, 2019). However, because the actual stimulus of each workout is largely dependent on the trainee’s discretion of effort, the HIFT strategy would seem to fall between “no-periodization” and “non-linear periodization” classifications. HIFT is not completely devoid of periodization structure (*i.e*., no periodization), but is not a true non-linear plan either. Whereas a non-linear structure will repeat a microcycle (over several weeks to months) that consists of modulated programming variables that target multiple, related physiological traits (Haff, 2015a; Haff, 2015b), HIFT aims at everything and does not repeat a specific pattern (Feito et al., 2018b; CrossFit, 2019).

### Rationale

Since the early 2010s, studies involving HIFT-related outcomes have grown rapidly (Feito, Brown & Olmos, 2019). Indeed, the scientific record has grown from less than 10 articles in 2012 to approximately 30 articles in 2015, though most of these limited their investigation to safety issues. In 2018, over 100 articles could be found, and that number doubled by 2021. While the growth of peer-reviewed evidence related to HIFT is encouraging and improves our understanding, 200 studies is a very small number for such a diverse programming strategy. Nevertheless, several recent systematic reviews have attempted to make sense of the risks and benefits associated with HIFT (Claudino et al., 2018; Meyer, Morrison & Zuniga, 2017; Rodriguez et al., 2021; de Souza et al., 2021; Schlegel, 2020; Jacob et al., 2020; Gean, Martin & Cassat, 2020; Barranco-Ruiz et al., 2020). Systematic reviews are often conducted to “*confirm or refute a current practice based on relevant evidence, establish the quality of the evidence, and to address variation in practice*” (Munn et al., 2018). However, the lack of comprehensive research on any given HIFT topic hinders this endeavor. Instead, a scoping review, which quantifies the volume of evidence on a specific topic and provides a broad or detailed overview of its focus (Munn et al., 2018), may be more appropriate at this time to summarize and synthesize current evidence. Since more evidence is available about acute responses than long-term training effects (Feito, Brown & Olmos, 2019) the aims of this scoping review are to (1) summarize currently available research related to HIFT and its effects on acute physiological outcomes, (2) identify research gaps, and (3) propose future research directions to continue to expand our knowledge of this training modality. The findings of this review are intended to inform scientists across a broad range of disciplines of the current standing of HIFT-related research and important methodological needs for future endeavors. Additionally, coaches, athletes, and healthy adults who participate in HIFT may find this review useful for better understanding the expected response to individual workouts.

## Methodology

With the goal of providing a broad scope of the literature related to acute physiological responses to HIFT, the present scoping review was completed in accordance with Arksey and O’Malley’s five-stage framework (Arksey & O’Malley, 2005). The following sections report this process.

### Stated research questions

Compared to the research endeavors of more traditional exercise strategies and sports, HIFT is a relatively new topic (Feito, Brown & Olmos, 2019). Programming relies on an extremely large number of possible exercise and workout structure combinations to elicit adaptations in several areas of fitness (the Level 1 Training Guide lists 10 fitness domains) (Feito et al., 2018b; CrossFit, 2019), and researchers have several objective and subjective means at their disposal to measure each of these areas. Thus, the following research questions were considered for this review: (1) *What outcome variables are most often studied in HIFT literature?* (2) *What are the acute physiological outcomes observed as a result of a single HIFT workout?*

### Identifying relevant studies

**Figure 1:**
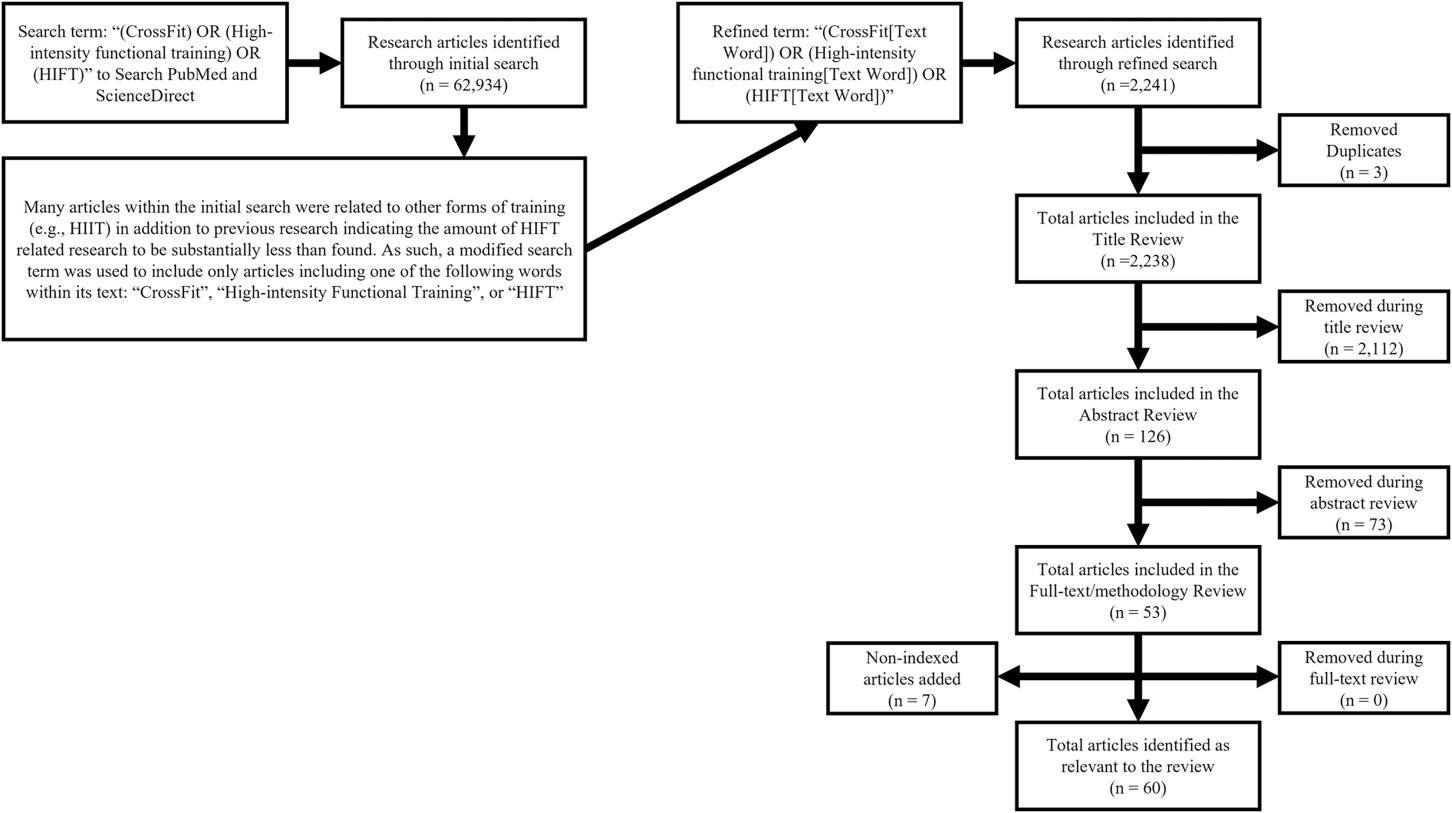
Multistage database review process.

CrossFit® Inc.’s Level 1 Training Guide proposes a theoretical template for constantly varied exercise where all modalities, physiological traits, and sport-relevant skills are sufficiently addressed over the course of training to help reduce the likelihood of neglecting specific health or fitness variables (*i.e*., those that might be neglected say, if an individual only focused on weightlifting or cardiovascular endurance) (CrossFit, 2019). This template shares many similar definitions and methodological similarities to those stated for HIFT (Feito et al., 2018b). Because of these similarities, as well as the sheer volume of CrossFit®-affiliated training facilities worldwide, CrossFit® participants and training facilities are commonly involved within HIFT-related research (Feito et al., 2018b; CrossFit, 2017). Thus, the search terms used for this scoping review included “(CrossFit) OR (High-intensity functional training) OR (HIFT)” in the following academic databases: PubMed and ScienceDirect. These databases alone were chosen as they are both free to the public and focus on the biomedical research that was most relevant to this review. However, the initial search revealed substantially more articles than indicated in previous research (Feito, Brown & Olmos, 2019), as such, the search was refined to “(CrossFit[Text Word])” OR “(High-intensity Functional Training[Text Word]) OR (HIFT[Text Word])”. The search was focused on peer-reviewed articles published between January 1^st^, 2000, and July 31^st^, 2022. These dates include the most current research and date back to the creation of CrossFit® in 2000 (CrossFit, 2019; CrossFit, 2017). As the most prominent form of HIFT, it is unlikely that any research was conducted on exercise strategies that fit the definitions of HIFT or CrossFit® prior to this date (Feito et al., 2018b; CrossFit, 2019). Finally, since the acute physiological responses to exercise are likely to be altered by various illnesses and age (specifically adolescent and older adult populations), only articles that included healthy, young adults were considered. Figure 1 illustrates the article search process and inclusion exclusion criteria are presented in Table 1.

**Table 1:** Inclusion and exclusion criteria.

Criterion	Inclusion	Exclusion
Time period	2000 to 2022	Studies outside of described time period
Language	English	Non-english studies
Types of articles	Original research published in a peer-reviewed journal	Case studies or series, reviews, non-original or non-peer-reviewed articles
Ethical clearance	Studies with approved ethical clearance	Studies without approved ethical clearance
Literature focus	Physiological outcomes related to CrossFit® or HIFT	All other acute or chronic outcome measures related to CrossFit® or HIFT
Population	Adults between the ages of 18 and 64	Non-human studies, ages outside of described age range, injured or clinical populations

### Study selection

The multi-stage selection process (see Fig. 1) required one author to initially review and identify articles of interest (JMM). Abstracts and Methods sections of each were reviewed to ensure that training sessions and outcome measures were in accordance with the stated definition of HIFT (Feito et al., 2018b) and included outcome measures immediately following an individual exercise session. Discrepancies were discussed by two authors (JMM and YF) and whenever disagreement occurred between these investigators, a third investigator was consulted (GTM). The decision to keep or discard an article was determined by the group after careful consideration of the study’s goals and the aims of this scoping review. Any study where a mutual agreement was not reached was eliminated from the sample (*n* = 0).

### Charting the data

The following information was extracted from the selected articles: author(s), year of publication, exercise protocols and key physiological findings. Descriptions of common HIFT workouts (*e.g*., benchmark and competition workouts) are available online (CrossFit, 2022; WODwell, 2022). Uncommon workouts (*i.e*., those specifically designed for research) are described in Table 2.

**Table 2:** Reported high-intensity functional training sessions.

First author, year	Structure and time	Movements
Brisebois (2014), Brisebois, Biggerstaff & Nichols (2021)	AMRAP 15:00	324 m run 10 × burpees 15 × kettlebell swings (20.5/11.4 kg)
Butcher, Judd & Benko (2015)	AMRAP Series 6 × 1:00 work and 3:00 rest	8 × bench presses (70% 1-RM) 10 × pull-ups (or ring rows) Box jumps (AMRAP)
Shaw et al. (2015)	AMRAP 10:00	3 × burpees 4 × push-ups 5 × air squats
Tibana et al. (2016)	AMRAP 10:00	30 × rope jumping (double-unders) 15 × power snatches (34 kg)
	AMRAP 12:00	250-m rowing 25 × burpees to 6″ target
Fisker et al. (2017)	5 Rounds for Time	5 × front squats (50/30 kg) 10 × box jumps (60/50 cm) 15 × rope jumping (double-unders)
Kliszczewicz et al. (2017)	AMRAP 15:00	250-m rowing 20 × kettlebell swings (16 kg) 15 × dumbbell thrusters (13.6 kg)
Mate-Munoz et al. (2017)	AMRAP 5:00	Power cleans (40% 1-RM)
Tibana et al. (2019a)	AMRAP Series 4 × 4:00 work and 2:00 rest	Round 1: 5 × thrusters (60 kg) 10 × box jump-overs
		Round 2: 10 × power cleans (60 kg) 20 × pull-ups
		Round 3: 20-calorie rowing 40 × wall ball shots (9 kg)
		Round 4: 15 × jerks (60 kg) 30 × toes-to-bar
Tibana et al. (2019b)	For Time (Team of 3)	500–1,000–1,500-m rowing 30–24–18 × handstand push-ups 15–12–9 × ring muscle-ups
	3 Rounds for Time (Team of 3)	15 × hang power cleans (40 kg) 20-m overhead lunges (40 kg) 25 × toes-to-bar 10 × jerks (40 kg)
	For Time (Team of 3)	27 × burpee box jump-overs 21 × legless rope climbs
	3 Rounds for Time (Team of 3)	15-m handstand walking 6–4–2–snatches (60–70–85 kg) 15-m handstand walking
	For Time (Team of 3)	30–40–50–60 calories on bike 20–16–12–10–thrusters (50–60–70–75 kg)
Timón et al. (2019)	AMRAP 5:00	1 (+1) × burpees 1 (+1) × toes-to-bar
	3 Rounds for Time	20 × wall ball shots (9 kg) 20 × power cleans (40% 1-RM)
Browne et al. (2020)	AMRAP Series Alternating between 1:00 Biking and 1:00 Functional Movement	Kettlebell high-pulls TRX pull-ups dumbbell front squats kettlebell suitcase squats TRX chin-ups dumbbell shoulder press kettlebell one-arm rows barbell biceps curls dumbbell push presses kettlebell reverse lunges TRX rows dumbbell squat presses kettlebell goblet squats TRX triceps extensions dumbbell overhead reverse lunges
Faelli et al. (2020)	AMRAP Series* 4:00 work and 2:00 rest	Round 1: running (rest) Rope jumping (single-unders) Round 2: pull-ups & air Squats (rest, repeat) Round 3: kettlebell swings & front squats (65–75% 1-RM) (rest, repeat)
Garnacho-Castaño et al. (2020)	AMRAP 8:00	1:30 wall ball shots (10 kg) 3:00 rest 1:00 back squats 3:00 rest 1:30 wall ball shots (10 kg) 1:00 back squats
Pearson et al. (2020)	AMRAP Series (8, 0:20 active periods with 10-s rest intervals) for each exercise	Rowing calories dumbbell thrusters (4.6 kg) Russian kettlebell swings (15.9 kg) burpees
Toledo et al. (2021)	AMRAP 20:00	13/11-calorie rowing 12 × deadlifts (62/44 kg) 10 × burpees over-the-bar 8 × kettlebell swings (24/16 kg)
	Rounds (equal to 20-min AMRAP) for Time	21/18-calorie rowing 18 × deadlifts (62/44 kg) 15 × burpees over-the-bar 12 × kettlebell swings (24/16 kg)

**Note:**

* The number of required repetitions were not reported.

## Results

After refining the search and removing article duplicates (*n* = 3), 2,238 articles remained. Of these, additional articles (*n* = 2,112) were removed because their title indicated that they did not specifically relate to HIFT or focus on healthy, young adult populations. Abstract and full-text review, focusing on methodology, led to the removal of another 73 and 0 articles, respectively. Throughout this process, a handful of known relevant articles (*n* = 7) were missed by the search and added to the final volume (Cavedon et al., 2020; Adami et al., 2021; Ahmad, Jusoh & Tengah, 2019; Babiash, 2013; Box et al., 2018; Brisebois, 2014; Brisebois, Biggerstaff & Nichols, 2021; Browne et al., 2020; Butcher, Judd & Benko, 2015; Carreker & Grosicki, 2020; Coco et al., 2019; Collins & Kearns, 2020; Durkalec-Michalski et al., 2018; Durkalec-Michalski et al., 2021; Escobar, Morales & Vandusseldorp, 2016; Escobar, Morales & Vandusseldorp, 2017; Faelli et al., 2020; Falk Neto et al., 2020; Feito et al., 2018a; Fernández et al., 2015; Fisker et al., 2017; Fogaça et al., 2020; Forte et al., 2022; García-Fernández et al., 2021; Garnacho-Castaño et al., 2020, 2022; Gomes et al., 2020; Kliszczewicz et al., 2017, 2018a, 2019, 2015, Kliszczewicz, Snarr & Esco, 2014; Kliszczewicz et al., 2018b, 2016; Kodikara, Walker & Wilson, 2021; Landers-Ramos et al., 2022; Leitão et al., 2021; Mangine et al., 2019, 2018; Maroufi et al., 2020; Martínez-Gómez et al., 2022; Maté-Muñoz et al., 2022, 2018, 2017; Morris et al., 2019; Pearson et al., 2020; Perciavalle et al., 2016; Schubert & Palumbo, 2019; Schwarz et al., 2020; Shaw et al., 2015; Tibana et al., 2016, 2018a, 2019a, 2018b, 2019b; Timón et al., 2019; Toledo et al., 2021; Wilke, 2020; Willis et al., 2019; Zecchin et al., 2021). The total remaining articles (*n* = 60) were organized into eight overarching categories based on their outcome measures (*e.g*., lactate, chronotropic, energy expenditure and oxygen consumption, hormonal, inflammatory, blood glucose, muscle and oxidative damage, and acute power output).

### Indicators of workout intensity

Different exercise modalities affect the method used to define a workout’s intensity. The intensity of most resistance training exercises is defined by the load being lifted and its relationship to either the maximum number of repetitions the trainee can complete with that specific load or the most amount of weight they can lift in the same movement for one repetition (Sheppard & Triplett, 2015). Though these methods are often used to quantify muscle strength, strength-endurance, and/or power, there are many exercises that are not amenable to this method. There is a greater influence from technique on maximal Olympic weightlifting loads compared to, say, the power lifts (*e.g*., back squats, deadlifts, and presses). Meanwhile, it is highly uncommon, too variable, or even unsafe to quantify maximal repetitions or loads for other exercises that fall into this category (*e.g*., assistance exercises, kettlebell swings, wall ball shots). Likewise, the intensity of many gymnastic movements prescribed during HIFT is not easily quantified. Individual body mass is often the load being lifted, and strength matters, but the trainee’s technical skill in performing the movement is extremely influential. Individuals who lack the skill or do not perform a specific movement efficiently will experience additional strain, put forth more effort, and complete more work (McGinnis, 2020). Since a HIFT workout may contain one or more of either of these modalities, researchers have often settled on quantifying intensity *via* traditional objective (*e.g*., lactate concentrations, heart rate, oxygen consumption) or subjective (*e.g*., ratings of perceived exertion) cardiorespiratory exercise metrics (Reuter & Jj, 2016). Though it is unknown how weightlifting intensity loads and gymnastic movement difficulty play into an overall workout’s intensity, using cardiorespiratory metrics seem to best summarize this characteristic. To maintain consistency with the scope of this review, this section will focus on objective, physiological indicators of HIFT workout intensity.

#### Blood lactate

During exercise, the point at which lactate begins accumulating faster than the body is able to remove, or clear it, is referred to as the lactate threshold (Faude, Kindermann & Meyer, 2009). The lactate threshold within trained and untrained populations is commonly associated with approximately 85% of one’s maximal heart rate (HRmax) and is described as the point at which exercise intensity progresses from moderate (*i.e*., below lactate threshold) to vigorous (*i.e*., greater than lactate threshold) (Foster, Fitzgerald & Spatz, 1999; Messonnier et al., 2013). Although the acute exercise bouts varied greatly, lactate concentrations immediately following exercise were either on par with, or even greater than that of commonly used maximal aerobic capacity (*i.e*., ≥8–10 millimoles per liter) and Wingate anaerobic (~13–15 millimoles per liter) testing criteria (Ahmad, Jusoh & Tengah, 2019; Babiash, 2013; Collins & Kearns, 2020; Escobar, Morales & Vandusseldorp, 2016, 2017; Falk Neto et al., 2020; Feito et al., 2018a; Fernández et al., 2015; Gomes et al., 2020; Kliszczewicz et al., 2017, 2018a, 2018b; Perciavalle et al., 2016; Tibana et al., 2016, 2018a, 2019a, 2018b; Timón et al., 2019; Toledo et al., 2021; Mate-Munoz et al., 2017, 2018; Öztürk, Özer & Gökçe, 1998; Vincent et al., 2004).

Of articles reporting lactate concentrations, two reported values outside of the expected range for vigorous exercise (Coco et al., 2019; Shaw et al., 2015). Shaw et al. (2015) observed a 170% increase in lactate concentrations, though raw concentrations (*i.e*., average pre- to post-exercise lactate increased from 2.20 to 5.95 millimoles per liter) were much lower than all other studies reviewed. Previous research indicates that a lower lactate response to exercise may be expected in untrained individuals compared to their trained counterparts of the same age group (Silverman & Mazzeo, 1996). Because greater lactate concentrations are expected with greater exercise intensities (Lagally et al., 2002), the lower concentrations observed by Shaw et al. (2015) are likely the consequence of a sedentary population with no HIFT experience being assigned low intensity exercises (*i.e*., burpees, push-ups, and bodyweight squats) for a relatively short duration (*i.e*., a 10-min AMRAP). A similar explanation might account for the slightly higher, but still lower than expected concentrations (*i.e*., peak of 6.34 millimoles per liter) reported by Coco et al. (2019) following a workout (CrossFit® Open workout 15.5) that included thrusters (95 lbs.) and calorie rowing. Though participants were considered professional bodybuilders, they were naïve to HIFT, and that lack of experience may have necessitated familiarization trials. This possibility remains unclear because the authors did not report workout performance, which could have been used to provide some indication of their percentile rank in the workout. For instance, the lower lactate concentrations combined with a higher percentile rank might have suggested that the workout did not represent a challenge. Afterall, they would likely have been accustomed to completing workouts involving several lifts using moderate-to-high volume loads for several sets and short rest intervals (Sheppard & Triplett, 2015; Ratamess et al., 2009). Unfortunately, a description of participant training history was also neglected in this study. Nevertheless, it seems reasonable to hypothesize that the lack of participant HIFT experience was responsible for the lower lactate concentrations observed in these two studies (Coco et al., 2019; Shaw et al., 2015) compared to others (Escobar, Morales & Vandusseldorp, 2016; Fernández et al., 2015; Kliszczewicz et al., 2017; Perciavalle et al., 2016; Tibana et al., 2018b). This idea is supported by differential lactate responses (*i.e*., 10 millimoles per liter) by less skilled participants who performed the workout “Fran” in a scaled manner (*i.e*., utilizing assistance during the pull-up repetitions) compared to a 50% greater elevation in lactate concentrations (*i.e*., +15 millimoles per liter) among those who performed the workout as prescribed (Babiash, 2013; Tibana et al., 2018b).

The remaining articles report lactate values reaching ≥13.3–18.9 millimoles per liter (~200–700% change) following eleven unique HIFT workouts (Fernández et al., 2015; Kliszczewicz et al., 2017; Perciavalle et al., 2016; Tibana et al., 2019a, 2018b; Timón et al., 2019; Toledo et al., 2021; Forte et al., 2022). All workouts included at least one weightlifting exercise except for the study by Fernández et al. (2015), which used the bodyweight workout “Cindy”. The greatest lactate responses were 17.8, 18.4, and 18.9 millimoles per liter following three workouts comprised of different modality combinations (*i.e*., weightlifting and gymnastic exercises, multiple weightlifting exercises, compared to a combination of weightlifting, gymnastics, and monostructural, respectively) and durations (*i.e*., 4.1, 8.9, and 20 min, respectively) (Tibana et al., 2019a, 2018b; Timón et al., 2019), which led to three different pacing (*i.e*., repetitions per second) strategies. Participants completed the shortest workout (*i.e*., “Fran”) at a pace of 0.36 repetitions per second, the 8.9-min workout at 0.22 repetitions per second, and the 20-min workout at 0.17 repetitions per second. These reports collectively (Fernández et al., 2015; Kliszczewicz et al., 2017; Perciavalle et al., 2016; Tibana et al., 2019a, 2018b; Timón et al., 2019; Toledo et al., 2021; Forte et al., 2022) suggest that, regardless of workout composition, higher lactate concentrations may be expected in experienced HIFT trainees than what would be expected from maximal testing in young, apparently healthy adults (Edvardsen, Hem & Anderssen, 2014).

#### Chronotropic responses

Thirty-one articles describing the chronotropic (or heart rate) response to HIFT were identified. Among these, exercise intensity loads ranged from “low” (*i.e*., bodyweight only) (Kliszczewicz, Snarr & Esco, 2014) to “high” (>100 kg) (Box et al., 2018) for short (Tibana et al., 2018b) (<5 min) to long (35 min) durations (Browne et al., 2020), and comprised of only a single (Kliszczewicz et al., 2017) to multiple (5+) exercises (Durkalec-Michalski et al., 2021). Despite large variations in workout programming characteristics, heart rate responses typically fell within the “vigorous intensity” category (*i.e*., between 77% and 95% of HRmax) (Liguori, 2021), though two investigations reported responses within the “light to moderate” range (*i.e*., <65% HRmax) (Shaw et al., 2015; Timón et al., 2019). These findings might be explained by the duration (5–10 min) of workouts entirely composed of low-intensity, low-complexity calisthenic exercises (*i.e*., burpees, push-ups, air squats, toes-to-bar). However, they contrast the heart rates (85–90% HRmax) reported by Butcher, Judd & Benko (2015) at 5 and 10 min into a 20-min workout of similar intensity-exercise composition (*i.e*., “Cindy”) in both novice and experienced trainees. It is possible that differences in participant HIFT experience, which varied from no experience and 6 months sedentary (Shaw et al., 2015) to 1–8 months for “novice” (Butcher, Judd & Benko, 2015) to >12 months (Timón et al., 2019) to >18 months for “experienced” (Butcher, Judd & Benko, 2015), could partially explain these discrepancies in conjunction with the pacing strategy allowed by workout composition. Novice (19.5 repetitions per minute) and experienced participants (23.5 repetitions per minute) averaged a faster pace over the 20-min workout completed in the study by Butcher, Judd & Benko (2015) than those in the 5 min workout (18.3 repetitions per minute) examined by Timón et al. (2019). In the latter study, the workout used an ascending repetition ladder scheme (1-1, 2-2, 3-3, …) for two exercises (burpees and toes-to-bar). Compared to the longer sets of “Cindy” (*i.e*., 5, 10, and 15 repetitions), more time must be spent transitioning between exercises during the early rounds, as few participants would have exceeded seven repetitions in any set (average AMRAP score was 91.4 repetitions) (Timón et al., 2019). While Shaw et al. (2015) did not report performance scores, the assigned triplet of three burpees, four push-ups, and five body squats would have also led to more frequent transitions and less fatigue on any set. Thus, the combination of experience, low-intensity/complexity programming, and slower pacing may explain the blunted heart rate responses seen in these two outlier studies.

Heart rate variability (HRV) is another metric that may be simultaneously collected while assessing the heart rate response to HIFT. As an indicator of autonomic control (Acharya et al., 2006), it is less relevant to a single workout’s intensity and more relevant to the trainee’s cumulative response to training. That is, whenever a trainee performs multiple workouts within a single training session or completes sessions on consecutive days, their autonomic response may be a useful indicator of readiness, which could impact the intensity of an upcoming workout. Thus far, three studies have reported the HRV response to HIFT (Kliszczewicz et al., 2018b; Kliszczewicz et al., 2016; Mangine et al., 2019); covering five unique workouts in total. As expected, root mean squares of successive normal-to-normal differences (RMSSD; reported as lnRMSSD) was depressed by nearly 50% following each workout (Kliszczewicz et al., 2018b; Kliszczewicz et al., 2016; Mangine et al., 2019), but methodological differences in post-exercise time points cloud any conclusions about recovery. For instance, lnRMSSD remained depressed for 1 h after “Cindy”, but because it was not tracked after 60 min, it is unknown when values returned to baseline (Kliszczewicz et al., 2016). In a later study, nearly identical patterns were noted following both “Grace” and a 15-min AMRAP comprised of rowing, kettlebell swings, and dumbbell thrusters (Kliszczewicz et al., 2018b). lnRMSSD remained depressed 45 min post-exercise and returned to baseline at 2 h, leaving whatever happened in the interim unknown. Likewise, Mangine et al. (2019) reported similar patterns where lnRMSSD remained depressed for 30 min following CrossFit® Open workouts 16.3 and 16.4. These two comparative studies suggest autonomic function may be expected to return to baseline within 45- and 120-min post-exercise following HIFT workouts. However, there are currently too many differences between studies to make this hypothesis. Kliszczewicz et al. (2018b) required participants to possess only 3 months of HIFT experience, complete all workouts in the morning, and in a laboratory setting. In contrast, Mangine et al. (2019) recruited participants with at least 2 years of HIFT experience and tracked HRV within a competitive setting at a time similar to when they normally trained. With the nature of competition (*i.e*., setting, opponent quality, provocation) being known to affect anxiety and the autonomic nervous system compared to normal training (Casto & Edwards, 2016; Kivlighan & Granger, 2006; Kraemer & Ratamess, 2005), it is clear that few fair comparisons can be made at this time. More research is needed using similar methods before generalized conclusions are possible.

### Biochemical responses

A limited number of studies have examined hormonal responses to HIFT (Faelli et al., 2020; Gomes et al., 2020; Kliszczewicz et al., 2018a; Kliszczewicz et al., 2018b; Kliszczewicz et al., 2016; Mangine et al., 2019; Mangine et al., 2018; Tibana et al., 2019b). Collectively, they report on changes in a handful of hormones following a variety of workouts within vaguely described populations. Thus, a major gap still exists in our understanding of both the acute and long-term effects of HIFT on human physiology. An acute bout of HIFT, like any workout, may be characterized by the manipulation of its programming variables (*e.g*., intensity, volume, density, *etc.*) to produce a stimulus that ultimately may lead to adaptations (Ratamess et al., 2009). An effective stimulus is one that exceeds the individual’s current ability to meet its imposed demands (Ratamess et al., 2009). Hormones respond to the stimulus to assist in meeting metabolic demands, restoring homeostasis, achieving steady state, and/or facilitating tissue repair. The extent of the response is dictated by a variety of factors that include, among others, the individual’s age, sex, fitness level, training status, nutritional and hydration status, and the nature (*e.g*., relative difficulty, novelty, context, *etc.*) of the training stimulus (Kraemer & Ratamess, 2005; Kraemer, Ratamess & Nindl, 2017). How each individual hormone responds only represents a portion of the complex, integrated response by the entire endocrine and related physiological systems (Kraemer & Ratamess, 2005; Kraemer, Ratamess & Nindl, 2017). In this regard, the current state of HIFT research has yet to reach its infancy. Still, there is enough information to begin forming basic expectations and pose questions for future research.

#### Catecholamines

The duration of a typical HIFT workout can be as short as <2 min to longer than 1 h, though most are shorter in duration. This means that glucose and glycogen will be the most prominent energy sources (Harris et al., 1976; Essen-Gustavsson & Tesch, 1990; Cairns, 2006). Since glucose and glycogen are limited in supply and are needed for functions other than workout performance, the initial hormone response primarily is aimed at maintaining blood glucose concentrations by facilitating lipolysis and blocking glucose’s entry into the cell (Kraemer & Ratamess, 2005; Kraemer, Ratamess & Nindl, 2017). The most immediate effect is accomplished by the fast-acting catecholamines, epinephrine (E) and norepinephrine (NE). Following HIFT workouts lasting 3–20 min, consistent elevations in E and NE have been observed immediately post-exercise before returning to pre-exercise concentrations within 1 h (Kliszczewicz et al., 2018b, 2016; Mangine et al., 2019). In the earliest of these studies (Kliszczewicz et al., 2016), well-trained men completed “Cindy” and a matched-duration (20 min) treadmill run. There was also an attempt to match intensity by having participants run at a pace equal to 85% maximal heart rate (*i.e*., the expected heart rate achieved in “Cindy” during pilot work), but ultimately, greater heart rates and perceived effort were noted throughout the HIFT workout. Accordingly, E and NE concentrations were 150% and 94% greater immediately following “Cindy” than concentrations following the treadmill bout, and they remained higher than treadmill bout concentrations for 1-h post-exercise. As of yet, it remains unclear whether these responses were due to the slight differences in exercising heart rate (“Cindy” = 93–98% maximal heart rate; treadmill = 89–94% maximal heart rate) or possibly the frequent changes in body position required by the HIFT workout (*i.e*., lowering to floor, jumping to pull-up bar, *etc.*) compared to the consistent body position of treadmill running (CrossFit, 2019). Regardless, this has been the only study to compare the catecholamine response during HIFT to a more traditional exercise modality.

Some important considerations for making conclusions about the hormone response to any HIFT workout include the individual’s ability to auto-regulate pace (*e.g*., rest breaks within a set, speed of transition between exercises, *etc.*), the modifications available for exercise intensity and complexity, and HIFT’s competitive aspects. In a pair of follow-up studies (Kliszczewicz et al., 2018b; Mangine et al., 2019), catecholamine responses were compared between two HIFT workouts completed in a laboratory setting (Kliszczewicz et al., 2018b) and two other workouts completed in a competitive setting (Mangine et al., 2019). Within the laboratory, similar elevations in E (~450%) and NE (~600%) were seen immediately following “Grace” (3.4 ± 1.0 min) and an AMRAP session containing multiple exercise modalities (15 min), and these returned to pre-exercise concentrations within an hour. Although the catecholamine responses to these markedly different workouts were the same, concentrations were 88–93% less than those previously reported after “Cindy” (Kliszczewicz et al., 2016). The most obvious explanation would be that two different studies used two different groups of people. Hormone responses are highly individualistic (Beaven, Gill & Cook, 2008; Alen et al., 1988; Jensen et al., 1991), and though each of these HIFT studies required similar experience (≥3 months of HIFT), there were different skill-based qualifications. The former study (Kliszczewicz et al., 2016) enrolled men and women who could complete at least 14 and 10 rounds of “Cindy”, respectively, and they ultimately averaged 21.5 rounds. The requirements for the later study (Kliszczewicz et al., 2018b) involved being able to row on an ergometer, perform kettlebell swings and dumbbell thrusters, and complete “Grace” within 5 min, and these men also outperformed this final inclusion requirement. Thus, there is little to go on to compare these two samples. Only anecdotal evidence exists about what is considered good for “Cindy” and the observed scores for “Grace” suggest that the sample was representative of the 40^th^ percentile in that specific workout (Mangine, Cebulla & Feito, 2018). Differences in workout duration and pacing could also be responsible. The highest concentrations were seen with the longest workout (*i.e*., “Cindy”), but the factors influencing the extent of the catecholamine response are not limited to exercise duration (Kraemer & Ratamess, 2005). Muscle activation, force of contraction, volume completed, and rest intervals also matter, and the similar responses seen with “Grace” and a longer (15-min AMRAP) workout do not support any obvious patterns except, possibly the degree to which workout density involved intermittent *vs*. continuous work (*i.e*., pace or repetitions per minute or second). “Cindy” does not require much space and the only necessary equipment is a bar to perform pull-ups. This provides a better opportunity for the individual to minimize time between exercises than say, the components of the 15-min AMRAP. Indeed, participants completed one repetition every 1.86 s during “Cindy” compared to every 3.28 s during the longer workout. Meanwhile, “Grace” does not involve exercise transitions, only auto-regulated breaks. Still, the slowest rate (*i.e*., one repetition every 6.88 s) was seen during “Grace”, and likely because 30 clean and jerks at higher intensity loads costs more energy than what was programmed in the other workouts.

In addition to regulating pace, participants in the most recent investigation on the catecholamine response to HIFT were given the choice of completing the prescribed (*i.e*., Rx) or scaled version of two workouts, and these were completed alongside other members of their normal training facility (Mangine et al., 2019). Immediately following CrossFit® Open workout 16.3, Mangine et al. (2019) observed an elevation in E (92 ± 113%) before it returned to pre-exercise concentrations within 30 min 1 week later, pre-exercise E and NE concentrations were 306% and 550% greater than the previous week, and these were further elevated 77% and 326% after 16.4 and remained elevated for an hour. Although greater duration and intensity are the most obvious reasons for 16.4’s elevated response (Kraemer & Ratamess, 2005), the relative difficulty of each workout would still have been dependent on the individual’s ability to perform specific movements (*i.e*., muscle-ups or handstand push-ups) or lift prescribed loads. This is further complicated because the physiological impact of scaling a routine for these reasons has yet to be investigated, and there is no commonly accepted method for equating different HIFT workouts. The only element that remains consistent across any HIFT workout is that completing work as quickly as possible is typically the objective, which then might be used as a surrogate for equating effort. Still, without knowing how effort and pacing might change with modified exercises and loads, using workout difficulty to explain these differences is heavily subjective. It is also important to acknowledge the potential impact from accumulated stress and fatigue leading up to the third and fourth workouts of a 5-week, five-workout competition (Casto & Edwards, 2016; Kivlighan & Granger, 2006; Kraemer & Ratamess, 2005). Unfortunately, the lack of a baseline or analysis surrounding the first two workouts eliminates the possibility of observing this effect. Nevertheless, the need to examine cumulative physiological effects of multi-week HIFT competitions is further highlighted by the observed E and NE concentrations being higher than those previously reported (Kliszczewicz et al., 2018b; Kliszczewicz et al., 2016).

Aside from the uncertainty introduced by workout variations and the multi-week competition, sample characteristics may yet again provide a valid explanation for why 16.3 and 16.4 elicited the highest catecholamine concentrations. These participants needed to have at least 2 years of HIFT experience and those who competed in Rx (*i.e*., completed workout as prescribed with no scaling) ranked between the 57^th^ and 85^th^ percentiles for 16.3, and between the 52^nd^ and 90^th^ percentiles for 16.4; rankings that also include three and four women athletes who elected to scale workouts 16.3 and 16.4, respectively (*i.e*., placing them below all Rx athletes). This was only the sixth year of the CrossFit® Open competition and little was (and still is) known about the factors that influence progression into subsequent rounds. Anecdotally, it is widely held that an athlete’s final ranking in a workout can be drastically improved by completing just a few additional repetitions or finishing it just a few seconds faster. Regardless of whether these provide an average-to-above average competitor with a realistic chance to progress in the competition, it may impact the importance they place on their performance. It is possible that anticipation or a degree of self-imposed pressure to succeed and/or outperform other members of their gym may have led to an elevated autonomic response (Casto & Edwards, 2016; Kivlighan & Granger, 2006; Kraemer & Ratamess, 2005; Mauger, 2014; Skorski et al., 2015) compared to what was seen in a non-competitive, laboratory setting (Kliszczewicz et al., 2018b; Kliszczewicz et al., 2016).

#### Anabolic and catabolic hormones

While catecholamines provide a more immediate effect on the mobilization of substrates, a more delayed effect, most relevant to sustained activity and recovery, may be expected from cortisol (C) and growth hormone (GH) (Kraemer & Ratamess, 2005; Kraemer, Ratamess & Nindl, 2017). Both are thought to assist in maintaining blood glucose concentrations by blocking its entry into muscle and facilitating lipolysis, but C will also promote protein catabolism for gluconeogenesis (Kraemer & Ratamess, 2005; Kraemer, Ratamess & Nindl, 2017); GH does not (Cameron et al., 1988; Fryburg, Gelfand & Barrett, 1991). Further, their effects are not immediately realized because C must first enter the cell and interact with its nucleus, and GH must bind to a GH-specific binding protein to extend its relatively short half-life and enhance its effect (Kraemer & Ratamess, 2005; Kraemer, Ratamess & Nindl, 2017). Otherwise, many of its effects particularly those related to muscle growth, are mediated by insulin-like growth factor 1 (IGF-1) (Roith et al., 2001; Mauras & Haymond, 2005; Walenkamp & Wit, 2007). Another relevant hormone, testosterone (T), also responds to exercise to help prepare skeletal muscle for physical activity, mediate skeletal muscle protein synthesis and repair, limit protein catabolism, replenish metabolic substrates, and restore neuromuscular function (Kraemer & Ratamess, 2005; Kraemer, Ratamess & Nindl, 2017). Like C, it is traditionally understood that T must enter the muscle cell and interact with the nucleus to exert its effects (Vingren et al., 2010; Sinha-Hikim et al., 2004), though this may not always be necessary to be influential (Estrada et al., 2003; Hamdi & Mutungi, 2010). Nevertheless, its interaction and/or competition with C throughout this process (Viau, 2002; Chen et al., 1997; Crowley & Matt, 1996) is of interest. When concentrations of C are greater than T, it is thought that the individual is in a more catabolic state whereas the reverse is considered more anabolic. Consequently, the testosterone-to-cortisol (TC) ratio is often used as a basic gauge of anabolic status and indicator of a positive or negative response to external stress (Adlercreutz et al., 1986).

Exercise protocols that produce higher concentrations of blood lactate (*e.g*., higher volume loads with shorter rest intervals) have been well documented to elicit greater responses from C and GH (Kraemer & Ratamess, 2005; Kraemer, Ratamess & Nindl, 2017). Although individual effort and self-regulation influence the stress of each workout, a typical HIFT session can be equated to a high-volume, short-rest resistance exercise session due to common programming directives (*i.e*., AMRAP and TTC). These essentially maximize workout session density and therefore, should elicit responses from C and GH. Indeed, all four of the studies that have investigated C within the context of HIFT have reported significant elevations (Faelli et al., 2020; Gomes et al., 2020; Mangine et al., 2018; Tibana et al., 2019b), as did the only study to monitor GH (Kliszczewicz et al., 2018a). Faelli et al. (2020) reported greater elevations in C following a 1-h, AMRAP-style HIFT session (+300%) compared to a decrease in C after a high-volume, short rest (*i.e*., 15RM, 1-min rest intervals) resistance exercise session (−25%). Then as expected, the response was diminished after 8 weeks of training using similar but progressive programming, though the HIFT protocol still elicited greater concentrations. However, these results should be viewed as preliminary. The participants were only described as having 1 year of resistance training or HIFT experience, the training sessions do not appear to have been equated in any way besides both being three 1-h sessions per week, and their practical relevance is questionable. For instance, while the resistance exercise program progressed intensity and volume regularly (every 2 weeks), it did not target muscle groups (besides abdominal exercises) on more than one session per week and was predominantly comprised of assistance exercises. At best, this design might be sufficient to stimulate adaptations for novice trainees but not those with moderate experience (Haff, 2015a; Sheppard & Triplett, 2015), as described by the authors. The suitability of the HIFT sessions were equally questionable due to monotonous programming and limited progression. Outside of the only progressive element (*i.e*., an increase in percent intensity load after 4 weeks), participants were simply instructed to complete ‘*as many repetitions as possible*’ of the exact same exercises, within the same time intervals, across all 8 weeks of training. This, by definition (Feito et al., 2018b; CrossFit, 2019), is not consistent with HIFT, which promotes constant variation in programming. Thus, it remains unclear as to whom these results are most applicable and whether they would hold up in a fair comparison.

In a later study examining the effect of training experience on the physiological response to “Cindy” (Gomes et al., 2020), members from the same training facility were split into experienced (28.5 ± 6.4 months) and novice (6.0 ± 1.5 months) groups, based on how long they had been participating in HIFT. Greater elevations in C were seen immediately following the workout and up to 30 min post-exercise in the experienced group, but no differences were seen in the heart rate achieved, changes in blood lactate concentrations, or the participants’ perceived effort. This contradicts the expectation of novice trainees being more sensitive to the workout’s stress due to their assumed lack of training and familiarity (Kraemer & Ratamess, 2005; Kraemer, Ratamess & Nindl, 2017). While the authors’ explanation that the additional work completed by the experienced group was responsible for the greater C response has merit, that work does not necessarily equate to a greater training stress. In fact, when perceived effort was made relevant to rounds completed (*i.e*., effort per round), the novice group reported working much harder. Furthermore, the experienced group possessed greater strength (in the deadlift), repeat sprinting ability (*via* yo-yo recovery test level 1), and lower body fat percentage. Each of these, in some capacity, provide evidence of each individual repetition and round representing a lesser percentage of the experienced group’s maximal capability (*i.e*., a lower physiological stress), which should equate to a reduced C response (Kraemer & Ratamess, 2005; Kraemer, Ratamess & Nindl, 2017). That is, it is possible that the amount of work completed by the experienced group was proportional to their physiological advantages over the novice group. However, this speculation is based upon an overall rating of effort divided by rounds completed, and is thus, an estimated average that also assumes consistent effort was given throughout the workout. The actual physiological cost of each round, and how this cost may have varied, changed, and/or accumulated from round-to-round, remains unknown.

Not knowing the actual progression of effort and stress across the workout leaves open the possibility for other factors to have been at work. For example, the experienced group may have benefited from further developed (or more practiced) skills related to pacing and movement efficiency (*e.g*., pace consistency, wasted movements, planned *vs*. unplanned rest, seamless transitions, *etc.*). A more strategic and efficient approach would artificially reduce the physiological cost of each repetition and round, and therefore allow a greater amount of work to be completed within the same duration. Alternatively, poorly devised strategies (*e.g*., pacing that is overly aggressive, conservative, or unplanned) can either hasten the onset of fatigue and/or limit performance (Mauger, 2014; Skorski et al., 2015). In either case, possessing a greater amount of training experience should provide adequate opportunities to improve fitness and learn strategies that guard against the premature onset of fatigue, but these cannot be assumed. Indeed, the “well-trained” men who completed “Cindy” for the study by Kliszczewicz et al. (2016) averaged 6.4 more rounds (or approximately 192 more repetitions) than the “experienced” trainees from Gomes et al. (2020), and they were only described as having at least 3 months of HIFT experience. Even Kliszczewicz, Snarr & Esco (2014) pilot sample, which had the same experience requirement but also included women, averaged 2.7 more rounds (or 81 more repetitions). All these suggest that HIFT experience alone is probably not sufficient to describe training status because it does not account for the quality of training or experiences and skills learned prior to HIFT. Therefore, without a more detailed description, making fair comparisons across HIFT samples and workouts is quite difficult.

Currently, cross-over designed studies appear to offer the best information about differential hormone responses. Within a series completed by Kliszczewicz et al. (2018a, 2019, 2018b) using 40^th^ percentile (in “Grace”) men, one study compared the responses of growth biomarkers (*i.e*., GH, IGF-1) following “Grace” and a 15-min AMRAP containing, low-intensity load exercises of varying modalities (Kliszczewicz et al., 2018a). Interestingly, despite being different in duration, both workouts produced similar elevations in blood lactate, but the GH response was greater, more rapid, and better sustained following the 15-min AMRAP. However, neither workout elicited a significant response from IGF-1 or its binding proteins (1 and 2). Typically, higher concentrations of blood lactate are accompanied by elevated GH and C (Kraemer & Ratamess, 2005; Kraemer, Ratamess & Nindl, 2017) to address the metabolic demands of exercise. Though it is odd that the GH responses were not consistent with associated lactate responses, pacing may have again been involved. Repetition completion rate during the longer workout was more than double that of “Grace”, and “Grace” lasted about one-third of the duration. Unfortunately, there is no way to contextualize these findings because no other studies have measured GH or IGF-1 in response to HIFT workouts.

The two remaining studies that examined hormonal responses to HIFT limited their focus to T and C responses within a competitive setting (Mangine et al., 2018; Tibana et al., 2019b). During the 2016 CrossFit® Open, Mangine et al. (2018) monitored changes in salivary concentrations of T, C, and TC prior to and following exercise on each week of the 5-week competition. Although T and C concentrations generally remained consistent prior to exercise, C concentrations were lower than all other weeks prior to exercise on week 5 (*i.e*., workout 16.5). Since this was the only workout that was completed at a nighttime “release event”, those values can likely be attributed to diurnal variations (Veldhuis et al., 1989). Still, TC remained steady prior to exercise on each week. This was an important observation because participants in this competition can repeat workouts as many times as needed to produce their best score within each week’s 4-day window, and they continued to participate in normal training throughout the week. The increased effort associated with competition and potentially higher volume load from repeated attempts over 5 weeks might be considered a period of overreaching or indicative of overtraining, as a large decline (~30%) in TC occurred (Adlercreutz et al., 1986). While that did not happen in these recreational athletes, monitoring the effect of training during competition on TC is still an important question that warrants investigation in more competitive populations.

The same investigation also noted similar elevations in C immediately following each workout, but different time courses for recovery (Mangine et al., 2018). A more rapid recovery was seen with the two shortest workouts (16.2 and 16.3), but this would have been expected (Kraemer & Ratamess, 2005). Meanwhile, T also responded to each workout except for the first week (16.1). This was interesting because T has been shown to respond to a multitude of program designs (Kraemer & Ratamess, 2005; Kraemer, Ratamess & Nindl, 2017), as long as “overload” is present (Ahtiainen et al., 2003; Hooper et al., 2017; McCaulley et al., 2009; Kraemer et al., 1990). Although 16.1 was completed as part of an actual competition, it is possible that the design of the workout lacked an “overloading” quality for those specific participants within the context of the study. It was the first workout of the competition and study, and data was only collected on each participant’s first attempt on any workout to limit the influence of familiarity on hormone responses. Moreover, several of the women in the study chose to complete the scaled version, which reduced the intensity load by ~50%. It is unknown how either load (*i.e*., Rx or scaled) compared to the participants’ maximal strength or whether scaling was even necessary, but the only usable indicator of relative difficulty was the C response and it, along with repetitions completed, did not appear to be affected by scaling. Without a more concrete metric of relative intensity or difficulty, the reason for the lack of a T response remains unclear. That said, relative intensity appears to be less of a consideration for designing HIFT workouts compared to maximizing workout density (*i.e*., completing work as fast as possible or maximizing repetitions in a specific time limit). In this regard, a typical or even competitive HIFT workout seems more likely to transiently affect C than T. This hypothesis was partially supported by Tibana et al. (2019b), who monitored T and C at 24-, 48-, and 72-h after a 3-day HIFT competition. The only changes observed were reductions in T (free and total) and C at 48-h with no effect on TC. Reasons for this reduction were not put forth and are difficult to ascertain from the information provided. The 3-day competition included five workouts that were completed by participants as members of 3-person teams. Though the contributions of team members on each workout were adequately described, the relative effort put forth and resultant performances were omitted. Additionally, the participants’ physical activity post-competition and diet (aside from maintaining “normal” diet) are not clear. Nevertheless, T and C returned to normal the following day, and these two investigations (Mangine et al., 2018; Tibana et al., 2019b) provide little indication that severe changes in anabolic status should be expected following a 3-day or 5-week HIFT competition.

#### Inflammatory markers

The mechanical and metabolic stresses experienced by skeletal muscle during exercise are known to trigger an inflammatory response both during exercise and the subsequent recovery period (Ellingsgaard, Hojman & Pedersen, 2019; Petersen & Pedersen, 2005). The implications of increased cytokine concentrations, however, are different depending on whether the inflammation is acute or chronic in nature (Petersen & Pedersen, 2005). As such, a broad understanding of the inflammatory cytokine cascade is needed, though an exhaustive review of the inflammatory response to exercise is beyond the scope of this analysis (for further information, see: (Petersen & Pedersen, 2005; Calle & Fernandez, 2010)).

Briefly, the inflammatory cytokine cascade is initiated with the release of the pro-inflammatory cytokines interleukin 1β (IL-1β) and tumor necrosis factor α (TNFα) (Petersen & Pedersen, 2005). These two cytokines will initiate the production and release of the pleiotropic cytokine, IL-6, which can be viewed as both a pro-inflammatory cytokine and an anti-inflammatory myokine (Ellingsgaard, Hojman & Pedersen, 2019; Petersen & Pedersen, 2005; Steensberg et al., 2000), which, in turn will result in increased concentrations of IL-10 and IL-1 receptor antagonist (IL-1ra) as well as a delayed increase in C-Reactive Protein (CRP) about 24 h later (Steensberg et al., 2003). IL-10 and IL-1ra will then suppress the actions of TNFα and IL-1β, aiding in the resolution of the overall inflammatory response (Petersen & Pedersen, 2005). The functions of CRP, however, are less clear as both pro- and anti-inflammatory actions have been linked to increased CRP concentrations, and the inflammatory profile is likely dependent on the predominant isoform in circulation (Sproston & Ashworth, 2018).

The source of IL-6 release appears to be the primary indicator of whether IL-6 functions in a pro- or anti-inflammatory role (Ellingsgaard, Hojman & Pedersen, 2019; Petersen & Pedersen, 2005). Though IL-6 has historically been viewed as a pro-inflammatory cytokine, within the context of exercise, IL-6 likely produces anti-inflammatory effects (Petersen & Pedersen, 2005; Pedersen & Bruunsgaard, 2003). IL-6 is released from skeletal muscle as a direct consequence of contraction (Steensberg et al., 2000), thus, within the context of the cytokine cascade, acute exercise likely bypasses the initial TNFα and IL-1β response, owing to the anti-inflammatory effects of exercise (Petersen & Pedersen, 2005). Moreover, the IL-6 response to exercise is augmented by glycogen depletion (Steensberg et al., 2001), and as such, exercise duration has historically been identified as the primary driver of IL-6 release (Fischer, 2006). Recent evidence, however, indicates a temporal relation between lactate and IL-6 concentrations (Hojman et al., 2019), indicating exercise intensity is also a stimulator of IL-6, though to a lesser extent (Fischer, 2006). Therefore, most forms of exercise should be expected to increase IL-6 concentrations, and consequently, all downstream cytokines and acute phase proteins. Despite this, increased circulating TNFα concentrations have been reported following resistance exercise resulting in muscle damage (Townsend et al., 2013; Wells et al., 2016), as well as following aerobic exercise in high ambient temperatures (Starkie et al., 2005). Thus, disruptions of tissue integrity or excessive increases in core body temperature may be necessary for pro-inflammatory cytokine release, though these are not consistent findings (Smith et al., 2000). To date, five investigations have examined inflammation resulting from acute bouts of HIFT (Faelli et al., 2020; Fogaça et al., 2020; Kliszczewicz et al., 2019; Tibana et al., 2016), repeated bouts of HIFT (Tibana et al., 2016, 2019b) and chronic HIFT (Faelli et al., 2020).

##### Pro-inflammatory cytokines

Three prior investigations have examined pro-inflammatory cytokines during and following HIFT sessions, with one examining the TNFα response (Kliszczewicz et al., 2019), and two examining IL-1β (Faelli et al., 2020; Tibana et al., 2019b). Kliszczewicz et al. (2019) and Tibana et al. (2019b) failed to demonstrate increases in either TNFα or IL-1β following acute bouts of HIFT or a 3 day competition, respectively. While some prior investigations have demonstrated increases in TNFα following exercise (Townsend et al., 2013; Wells et al., 2016; Starkie et al., 2005), these studies are in highly trained individuals, completing a large volume of exercise that resulted in tissue damage (Townsend et al., 2013; Wells et al., 2016) or were completed in high ambient temperatures (Starkie et al., 2005). Given that Kliszczewicz et al. (2019) completed a relatively lower volume of overall exercise, and did not report markers of muscle damage, the failure to demonstrate changes in TNFα concentrations is not surprising (Petersen & Pedersen, 2005).

Investigations examining IL-1β in circulation have demonstrated largely inconsistent findings. Prior work has demonstrated significant increases following a marathon (Ostrowski et al., 1999), lower body plyometrics (Chatzinikolaou et al., 2010), a soccer match (Ispirlidis et al., 2008), and 45 min of running (Vassilakopoulos et al., 2003), while others have shown no change in IL-1β concentrations following resistance exercise (Buford, Cooke & Willoughby, 2009), 60 min of running (Scott et al., 2011), or long distance running (Nielsen et al., 2016; Ostrowski et al., 1998), whereas others have observed decreases in IL-1β concentrations following skeletal muscle damage (Smith et al., 2000). The inconsistency observed between studies is likely related to the low concentrations observed in circulation, which is commonly undetectable (Chatzinikolaou et al., 2010; Ispirlidis et al., 2008; Scott et al., 2011), as well as the previously suggested high rate of clearance from circulation (Ostrowski et al., 1999). Therefore, it is unclear whether the lack of change in IL-1β concentrations observed by Tibana et al. (2019b) is expected.

Faelli et al. (2020), however, observed an acute decrease in salivary IL-1β concentrations both prior to and following 8 weeks of HIFT training, with the decrease greater following training compared to prior to training. To our knowledge, only one other investigation examined the salivary IL-1β response to exercise, demonstrating an increase in salivary IL-1β secretion rate following 60 min of recumbent cycling at 75% VO2max (Usui et al., 2011). While these results appear to be opposing, it is important to distinguish between salivary concentrations and secretion rates. Changes in sympathetic activity are known to influence salivary production (Proctor & Carpenter, 2007), and thus can have a concentrating or diluting effect on salivary markers, depending on the level of sympathetic involvement. As such, comparisons that are not corrected for the expected changes in salivary flow rate following exercise (Chicharro et al., 1998) are difficult to interpret and may be more related to changes in salivary production than the marker of interest. Therefore, the decreases reported by Faelli et al. (2020) may have been related to changes in the salivary response to HIFT, rather than the IL-1β response to HIFT.

##### Anti-inflammatory cytokines and acute phase proteins

Two investigations have examined the IL-6 response to HIFT. The first, completed by Tibana et al. (2016) in 2016 examined the IL-6 response to two consecutive days of HIFT, and demonstrated the expected rise from pre- to post-exercise, with no differences between consecutive days of training (Tibana et al., 2016; Petersen & Pedersen, 2005). Kliszczewicz et al. (2019) examined the differences in IL-6 response to “short” (30 power clean & jerk with 61.4 kg; “Grace”) and “long” (15 min AMRAP; 250 m row, 20 kettlebell swings with 16 kg, 15 dumbbell thrusters with 13.5 kg) HIFT sessions; demonstrating greater IL-6 concentrations following the “short” exercise bout, though no time dependent changes were reported. It is important to note that the significant trial effect may not have been related to the exercise sessions themselves, as the statistical comparison between pre-exercise values also approached significance (*p* = 0.057). Notwithstanding, it is surprising that Kliszczewicz et al. (2019) failed to observe time dependent changes in IL-6 concentrations following either HIFT bout given the wide range of exercises that have been previously shown to stimulate IL-6 release, including a single Wingate (Abedelmalek et al., 2013), 5 × 3 min high intensity interval exercise (Croft et al., 2009), resistance exercise (Nieman et al., 2004) and continuous aerobic exercise (Nieman, Sha & Pappan, 2017). Importantly, however, the resting IL-6 concentrations reported by Kliszczewicz et al. (2019) were 4–5 fold higher than those typically reported in other exercise research (Abedelmalek et al., 2013; Croft et al., 2009; Nieman et al., 2004; Nieman, Sha & Pappan, 2017) as well as the concentrations reported by Tibana et al. (2016). Regardless, the 2–3 fold increase in circulating IL-6 observed by Tibana et al. (2016) is drastically lower than the 5–10 fold increase or greater that is typically associated with prolonged aerobic exercise (Fischer, 2006) and should be considered in this context.

Four investigations have examined the acute phase protein CRP (Fogaça et al., 2020; Tibana et al., 2019b) or the anti-inflammatory cytokine IL-10 (Kliszczewicz et al., 2019; Tibana et al., 2016, 2019b) in response to HIFT. Given the delayed response of CRP (Steensberg et al., 2003) and its predominance as a marker of chronic inflammation, it is not surprising that no changes were observed in CRP concentration following a single HIFT session (Fogaça et al., 2020). While it may be more expected to observe an increase in CRP concentrations following multiple days of HIFT competition, as was used by Tibana et al. (2019b), no changes were observed. Given that IL-6 will stimulate CRP, along with IL-10 (Steensberg et al., 2003), the failure of Tibana et al. (2019b) to observe a rise in IL-10 following 3 days of training, or on the second day of consecutive HIFT sessions (Tibana et al., 2016), it is possible the IL-6 response to HIFT is insufficient to produce an observable increase in either CRP or IL-10. Despite this, IL-10, was demonstrated to increase following a single bout of HIFT (Tibana et al., 2016), though others failed to show this response (Kliszczewicz et al., 2019). Importantly, both Tibana et al. (2016) and Kliszczewicz et al. (2019) assessed both IL-10 and IL-6. When time dependent increases in IL-10 concentrations were observed, time dependent increases in IL-6 concentrations were also observed (Kliszczewicz et al., 2019; Tibana et al., 2016).

##### Changes in immune parameters

The immune system is a complex integration of various cells, proteins and antibodies functioning together to protect the host from pathogens, as well as to aid in tissue repair following damage (Parkin & Cohen, 2001; Tidball & Villalta, 2010). The simplest form of immune measurement is the circulating counts of leukocytes and their subsets: lymphocytes, monocytes and granulocytes. The robust mobilization of leukocytes into circulation following exercise is well documented (Gleeson, 2007; Peake et al., 2017; Pedersen & Toft, 2000) and is primarily due to a neutrophilia and lymphocytosis (Robson et al., 1999; Nieman et al., 1994). The neutrophils and lymphocytes entering circulation as a result of exercise are primarily sourced from the marginal pool in response to increased sheer stress and epinephrine (Dimitrov, Lange & Born, 2010; Foster et al., 1986). Subsequently, a reduction of lymphocyte counts from approximately 30 min until up to 72 h after exercise results in a period of time the host may be vulnerable to opportunistic infections; the so-called ‘Open-Window’ (Nieman et al., 1994; Nieman, 1994; Simpson et al., 2020). Though increased rates of upper respiratory illnesses have previously been demonstrated following singular athletic events (Nieman et al., 1990; Peters, Shaik & Kleinveldt, 2010) and training (Spence et al., 2007; Gleeson et al., 2013), the post-exercise decline in lymphocyte counts has also been suggested to be programmed egress from circulation, triggered by cortisol (Okutsu et al., 2005; Okutsu et al., 2008), that enhances immunosurveillance (Simpson et al., 2020; Campbell & Turner, 2018). As such, there is no consensus as to whether exercise induced immunosuppression occurs (for a review, see (Simpson et al., 2020)), though immunosuppression is still considered a hallmark of over-reaching and overtraining syndrome (Meeusen et al., 2013).

##### Leukocytes

Two prior investigations have examined the leukocyte response to acute HIFT sessions (Durkalec-Michalski et al., 2021; Gomes et al., 2020) and demonstrated largely consistent findings with prior literature from other exercise interventions (Robson et al., 1999; Nieman et al., 1994; Arroyo et al., 2022; Kakanis et al., 2010). Briefly, Durkalec-Michalski et al. (2021) examined the reliability of the leukocyte response to an 18 min HIFT session (“Fight Gone Bad”) demonstrating reliable total leukocyte, lymphocyte and granulocyte responses across three training sessions among individuals experienced with HIFT. Moreover, the expected mobilization immediately following the HIFT session was observed for total leukocytes, lymphocytes and monocytes, though, the expected mobilization of granulocytes was not (*p* = 0.070) (Durkalec-Michalski et al., 2021). Gomes et al. (2020) demonstrated similar findings following a 20-min HIFT session (“Cindy”) in both individuals that were experienced and novice with HIFT. Briefly, both experienced and novice individuals demonstrated significant increases in total leukocyte and leukocyte subset counts immediately after exercise, though at 30 min post-exercise both groups observed suppressed lymphocyte counts relative to baseline, before returning to baseline levels 24 h later (Gomes et al., 2020). This finding is consistent with almost all other research, which has demonstrated a reduced lymphocyte count between 30 and 120 min post exercise following high intensity interval cycling (Arroyo et al., 2022), continuous aerobic cycling (Robson et al., 1999; Arroyo et al., 2022; Kakanis et al., 2010) and resistance exercise (Nieman et al., 1995). Therefore, changes in leukocyte populations are similar following HIFT when compared to other forms of exercise, likely due to the observed increase in cortisol concentrations that accompany HIFT (Gomes et al., 2020; Okutsu et al., 2005; Okutsu et al., 2008; Suzuki et al., 1996).

Though cortisol stimulates egress of lymphocytes and monocytes from circulation (Okutsu et al., 2005; Okutsu et al., 2008), it is also known to indirectly stimulate neutrophil production from the bone marrow, owing to the sustained rise in granulocyte counts following exercise (Suzuki et al., 1996; Davis et al., 1991). Interestingly, Gomes et al. (2020) reported a lower granulocyte count in novice when compared to experienced individuals 30 min after exercise, which may be attributable to the greater cortisol response to ‘Cindy’ in the experienced group (Davis et al., 1991).

##### Immunoglobulins

Immunoglobulins (Ig), commonly referred to as antibodies, are glycoproteins that are responsible for antigen recognition and are grouped into five primary classes based on their heavy chains; IgA, IgD, IgE, IgG and IgM (Júnior et al., 2010). The most commonly studied Ig is IgA, though it is most associated with saliva in exercise immunology literature due to its role in mucosal immunity (Bermon et al., 2017). Despite this, to date the only paper to have examined IgA following HIFT was collected from circulation (Tibana et al., 2019b). As such, interpretations from this data must be put into the proper context of the circulating IgA response, rather than the secretory IgA (SIgA) response.

Tibana et al. (2019b) demonstrated a small (~1–2%) increase in circulating IgA at 24 and 72 h following a 3 day HIFT competition, though no change in IgA concentration was present at 48 h post exercise. Given the minimal increases observed (Tibana et al., 2019b), and the failure of other exercise interventions to produce changes in circulating IgA concentrations (Peters, Shaik & Kleinveldt, 2010; McKune et al., 2005), these results are likely spurious. Importantly, the use of salivary IgA measurements are considered preferable (Bermon et al., 2017), likely due to the sensitivity of SIgA in response to exercise, and the previously reported changes associated with exercise (Peake et al., 2017; Bermon et al., 2017; Neville, Gleeson & Folland, 2008).

Collectively, most markers of inflammation and immune function respond similarly to HIFT as they do to most other types of exercise, however, certain markers require further investigation. Given the possible link between muscle damage and circulating TNFα concentrations (Townsend et al., 2013; Wells et al., 2016), future studies should examine the acute TNFα response to HIFT protocols that also result in significant muscle damage. Moreover, studies should focus on this response in untrained *vs*. highly trained individuals. Additionally, given the link between IL-6, IL-10 and CRP, future investigations examining the acute inflammatory response to HIFT should focus on IL-6; particularly using protocols examining differences between high volumes of aerobic workloads and anaerobic workloads to further elucidate the roles of lactate and glycogen in the overall IL-6 response. Furthermore, utilizing protocols with differing volumes of aerobic and anaerobic workloads should provide a greater distribution of the IL-6 response, which will aid in determining whether HIFT produces a sufficient increase in IL-6 concentrations to augment the IL-10 and CRP responses.

Investigations examining chronic HIFT should focus on the response of CRP and SIgA. Given the delayed response of CRP to inflammatory mediators (Steensberg et al., 2003), its overall role in the inflammatory process (Petersen & Pedersen, 2005), and its presence in chronic low-grade inflammation (Petersen & Pedersen, 2005; Lasselin & Capuron, 2014), CRP may offer an interesting avenue to monitor the chronic inflammatory response to training. Moreover, given the prevalence of elevated CRP concentrations in various clinical populations (Lasselin & Capuron, 2014), CRP also affords the ability to monitor the capacity of HIFT to reduce inflammation within chronic inflammatory diseases. Lastly, SIgA is considered a primary indicator of mucosal immunity (Bermon et al., 2017), and has previously been linked to increased rates of upper respiratory illnesses as well as training volumes (Peake et al., 2017; Bermon et al., 2017; Neville, Gleeson & Folland, 2008). As such, future investigations should examine how differing volumes of chronic HIFT may impact SIgA and the athletes’ susceptibility to upper respiratory illnesses.

#### Markers of damage

Repeated, strenuous muscle contractions result in ultrastructural damage to the muscle fiber membrane resulting in the leakage of intracellular components (*e.g*., creatine kinase (CK), myoglobin, lactate dehydrogenase) into circulation (Clarkson & Hubal, 2002). In recent years, HIFT has been perceived as an exercise regimen which produces potentially unsafe levels of muscular damage due to the high volume of work performed with moderate intensities. Indeed, several case reports have been published describing patients experiencing high levels of indirect markers of muscle damage and symptoms of rhabdomyolysis following HIFT style training (Doughty, 2017; Hopkins et al., 2019; Meyer, Sundaram & Schafhalter-Zoppoth, 2021). However, more recent studies and reviews consisting of large-scale surveys demonstrate that HIFT participants experience similar rates of rhabdomyolysis and other injuries as traditional resistance training and other exercise modalities (Dominski et al., 2019; Feito, Burrows & Tabb, 2018; Klimek et al., 2018). To develop a full picture of HIFT induced muscular damage, additional studies with robust injury epidemiological designs are warranted to provide appropriate metrics (*e.g*., epidemiologic incidence proportion, incidence rate, clinical incidence) of the likelihood of injury while participating in HIFT (Knowles, Marshall & Guskiewicz, 2006).

Within this scoping review, six Randomized Controlled Trials (RCTs) were evaluated which directly studied indirect markers of muscle damage following various HIFT protocols. Fogaça et al. (2020) implemented a HIFT workout consisting partially of heavy snatches (75–80% one-repetition maximum; 1RM) and AMRAP double-unders and power snatches yielded CK elevations immediately (+39%; 440.5 U/L) and 24 h post-exercise (+195.1%; 938.65 U/L) similar to values seen following high-volume resistance exercise (Gonzalez et al., 2014) with no significant elevations in subjective ratings of DOMS. However, most HIFT RCTs enrolling HIFT-trained individuals have reported small but significant increases in markers of muscle damage (Durkalec-Michalski et al., 2018; Durkalec-Michalski et al., 2021; Tibana et al., 2022), likely indicating that HIFT trained participants adapt to this style of training and experience diminished muscle damage responses over time. In the same vein, one study compared novice (3–8 months CrossFit® experience) to experienced (≥18 months CrossFit® Experience) and found no differences in CK values at any time point following the WOD “Cindy” (Gomes et al., 2020). There is a need in future studies to investigate markers of muscle damage in truly HIFT untrained or naïve individuals to outline the extent of muscle damage and timeline of functional recovery given the concerns raised by previous case studies. In comparison to other exercise modalities, Durkalec-Michalski et al. (2018) compared the muscle damage response between a HIFT workout (“Fight Gone Bad”) and an incremental aerobic cycling test. Although the HIFT workout consisted of resistance exercises and explosive movements (*e.g*., deadlift, push press, box jumps), there were no significant differences between conditions with both workouts producing small elevations of CK (+12.3%) and lactate dehydrogenase (+13.4%) immediately-post exercise likely attributed to uncorrected plasma volume shifts. Additionally, CK values following muscle damaging exercise typically peak 24–48 h post-exercise suggesting this study was not adequately designed to compare muscle damage between groups and highlighting the need for plasma volume corrections in future HIFT investigations examining blood markers. One unique study examined the influence of a 3-day CrossFit® competition consisting of six total workouts on markers of muscle damage in nine men (Tibana et al., 2019b). Creatine kinase values peaked at 24 h following the 3-day competition (+48%; 698.7 U/L), with levels returning below baseline values by 72 h after the competition. Interestingly, the authors note that resting CK values were elevated in this cohort (~472 U/L) potentially indicating incomplete recovery from workouts leading up to the competition. Thus, future work examining pre-competition recovery and tapering practices in HIFT athletes may be useful as the unique demands of the sport (*e.g*., resistance, plyometric, anaerobic, & aerobic exercise) poses a challenge to athletes attempting to optimize rest while preserving performance gains. Another important consideration is how HIFT sessions will typically place a HIFT-style “workout-of-the-day” after trainees complete a more traditional-style resistance training workout. Therefore, the true volume and intensity of weight lifted, and subsequent muscle damage, during an entire HIFT session may be much higher in some cases than the single “workout-of-the-day”, which have been studied in RCTs to date. Furthermore, given the diverse metabolic and muscular demand of the wide array of potential designs, future studies comparing the damage response to different HIFT sessions and workouts is also recommended.

#### Markers of oxidative stress

Oxidative stress manifests when the accumulation of reactive oxygen species surpasses the organism’s capacity to neutralize these free radicals by its antioxidant defense system (Urso & Clarkson, 2003). The excessive production of these byproducts, often by vigorous anaerobic and aerobic exercise, can promote cellular damage to lipids, proteins, and DNA (Nikolaidis et al., 2008). As described earlier in this review, HIFT provokes a metabolic stress and muscular damage, presumably initiating oxidative imbalances and thereby oxidative stress in its participants. To date, only two studies have investigated indirect markers of oxidative balance pertaining to HIFT programming. Kliszczewicz et al. (2015) examined the oxidative stress response of the workout “Cindy” to a high-intensity treadmill run. This metabolically taxing workout increased lipid peroxides at 1-h (+143%) and 2-h (+256%) post-exercise compared to pre-exercise values with no change in protein carbonyls and no differences observed between the workouts for any stress marker. The authors speculate that the exercise intensity (~90% HRmax) promoted the increase in lipid peroxides as both intense aerobic and anaerobic exercise has been shown to increase lipid damage (Bloomer et al., 2005). Furthermore, an increase in markers of antioxidant defense were seen for total antioxidant capacity and ferric reducing antioxidant power at 1-h and 2-h in both conditions (Kliszczewicz et al., 2015). Faelli et al. (2020) is the only other study observing HIFT induced oxidative stress, measuring salivary uric acid as an indicator of the body’s antioxidant defense. Twenty trained men were allocated to a CrossFit® or resistance training program for 8 weeks with saliva samples collected before and after the first and last workout in each respective program. Results showed significant increases in salivary uric acid following both the CrossFit® and resistance training sessions before and after the 8-week program with no differential responses between groups. However, a lessened percent increase in post-training uric acid levels were observed in the resistance training group, while the CrossFit® group experienced similar percent increases as the first exercise bout. Differences in the final workout programming may account for differences between the antioxidant defense response as the CrossFit® workout was designed as an AMRAP before and after training while the resistance exercise group began with a higher repetition range (15 reps @ 50%1RM) and progressed to a higher intensity (8 reps @ 75%1RM). Thus, due to the nature of HIFT workouts (*e.g*. AMRAP, very short rest periods, %HRmax) it is likely these workouts produce a constant challenge to the oxidant balance of the athlete with antioxidant defense markers (Uric Acid, total antioxidant capacity, ferric reducing antioxidant power) elevated to combat free radicals similar to Kliszczewicz et al. (2015). Clearly, more data is needed to make any meaningful conclusions regarding the magnitude of oxidative stress experienced during HIFT. Future studies should expand the timeframe of measurement as studies investigating eccentric or dynamic muscle damaging exercises commonly see elevations in blood markers of oxidative stress activity later in recovery (24, 48, 72 h) (Nikolaidis et al., 2008).

### Energy expenditure

Weight management through exercise is frequently sought for both aesthetic reasons and to combat complications related to obesity (Egli et al., 2011). For realistic and sustainable weight loss, or to prevent weight gain, the American College of Sports Medicine recommends that workout sessions should require approximately 300–400 kcal to complete for a total of 1,200–2,000 kcal per week (Donnelly et al., 2009). Provided, of course, the individual is also appropriately modifying caloric intake while still meeting nutritional needs (Seagle et al., 2009). Although the design of this strategy is simple, successful implementation is not a guarantee. Individuals often cite a lack of time and motivation as being the greatest challenges to meeting these recommendations (Hickey & Mason, 2017). These specific challenges (time and motivation) also represent some of the attractive aspects about HIFT. Facilities typically schedule classes in 1-h blocks that are divided into periods for warming up, possibly resistance training or gymnastic skill practice, a workout-of-the-day, and a cool-down period. The “workout-of-the-day” is the signature characteristic of HIFT sessions, and these rarely last more than 20 min. Indeed, across all studies examined in this review, these workouts lasted between 4 and 35 min. When compared to moderate-intensity exercise training, the shorter training duration may help alleviate lack of time as a barrier to exercise and promote continuation/adherence. For instance, obese, sedentary adults assigned to a HIFT-based exercise intervention exercised for significantly less time daily (and total time weekly) compared to the moderate-intensity group, and were reportedly more likely to continue their exercise regimen following the investigation (Heinrich et al., 2014). Additionally, high-intensity exercise has previously been reported as having similar and even greater physiological improvements related to energy expenditure (EE) and oxygen consumption compared to other exercise modalities (Tabata, 2019). When comparing a Tabata protocol (*i.e*., 20 s of exhaustive cycling followed by 10 s of rest for 7 to 8 sets) to continuous aerobic training at 70% VO2max, both strategies improved aerobic capacity over 6 weeks of training, but only the Tabata group experienced improved anaerobic capacity (Tabata et al., 1996). Finally, classes are usually held in a group setting. Group exercise may be more effective for sustaining adherence to training because, compared to training alone or with a personal trainer, they involve greater amounts of social recognition, competition, and social support (Heinrich et al., 2014; Hickey & Mason, 2017; Kanamori, Takamiya & Inoue, 2015). Accordingly, data suggests EE may be greater when completing the same workout in a group setting *vs*. training alone (Okonkwo, 2012). That said, by definition, the design of each HIFT session varies greatly from day to day, and the number of studies to examine EE during various workout designs and populations is negligible. Their consistency is largely unknown. Therefore, the purpose of this section is to establish a base for future research on describing EE in relation to HIFT.

#### Oxygen consumption

Eight studies have reported on the energy demands of HIFT (Babiash, 2013; Brisebois, 2014; Brisebois, Biggerstaff & Nichols, 2021; Browne et al., 2020; Fernández et al., 2015; Kliszczewicz, Snarr & Esco, 2014; Schubert & Palumbo, 2019; Willis et al., 2019). Of these, five reported an average EE of approximately 225 kcal following individual HIFT workouts (Babiash, 2013; Browne et al., 2020; Fernández et al., 2015; Kliszczewicz, Snarr & Esco, 2014; Willis et al., 2019), whereas three reported an average EE of 502 kcal for an entire hour-long HIFT class session (Brisebois, 2014; Brisebois, Biggerstaff & Nichols, 2021; Schubert & Palumbo, 2019). As expected, EE during HIFT appears to be heavily influenced by exercise duration (*i.e*., <10 min = ~60–170 kcal, 15 min = ~175–200 kcal, 20 min = ~260–320 kcal, and 35 min = ~465–580, complete HIFT session = ~460–605). Although the EE of an individual workout may help improve understanding the physiological demands and develop potential strategies for improving performance, EE from entire class sessions may be more useful for better understanding the effect of HIFT for weight management. Given the current evidence, it appears HIFT may be a viable and effective means of weight management (Donnelly et al., 2009). However, among studies examining EE and HIFT, several limitations exist in its measurement.

EE is frequently analyzed by measuring the oxygen demands of exercise, but this method is limited to that of the aerobic system and does not incorporate anaerobic pathways (*i.e*., phosphagen and glycolytic systems) (Scott, 2005; Matarese, 1997). This is problematic for accurately measuring EE when you consider the strategy’s high demand on anaerobic pathways (Scott, 2005), as evidenced by the high lactate concentrations that have been reported (see earlier section). Beyond that, measuring oxygen consumption during many HIFT workouts presents a logistical challenge when using traditional stationary and portable gas analyzers. There are several common examples within this strategy where these devices would interfere with performance (*e.g*., weightlifting exercises that might require the trainee to lift a bar from the ground to overhead, performing gymnastics on a pull-up bar or rings). Attempts have been made to counteract some of these difficulties, as one study utilized data from a graded exercise aerobic capacity test to create a regression equation that estimated oxygen consumption *via* heart rate (Babiash, 2013). Though this concept alleviates the physical constraints that negatively impact one’s ability to measure oxygen consumption during HIFT, the results were less than ideal. The data was drastically different from direct oxygen consumption *via* a portable gas analyzer (*e.g*., COSMED K4) and still did not account for anaerobic energy expenditure (Fernández et al., 2015). The regression data indicated that 88.2 kcal were burned during “Fran”, whereas direct oxygen estimated 121 kcal (Babiash, 2013; Fernández et al., 2015). With only one of those studies quantifying “Fran” TTC and comparing sex differences (Babiash, 2013), it remains unclear how body mass, lean mass, exercise pacing, and duration affected EE. More importantly, this comparison is only relevant to a single workout and between participants with unclear training histories; only one study reported experience (Fernández et al., 2015).

Data quantifying EE in relation to different HIFT workouts, or workout types, is clearly needed before accurate estimation of an entire class is possible. The unlimited potential for designing unique HIFT workouts does present a challenge, but a universal system for equating or classifying workouts (*e.g*., based off duration, complexity, intensity, or pacing) would help in this regard and alleviate the need to assess EE following every single workout in all populations. The lack of a non-invasive, but accurate, method for quantifying aerobic and anaerobic contributions to EE will continue to limit understanding on this topic. However, limiting between-laboratory differences in measurement techniques, descriptions of participant characteristics and training history, and in-study workout performance descriptions would also help. It is possible that better control over the factors known to influence EE would improve the accuracy of estimation equations.

#### Blood glucose

As discussed earlier, the maintenance of blood glucose during exercise is augmented by hormonal factors, the autonomic nervous system, as well as enzyme activity at the cellular level (Brooks et al., 2005). Because these and a variety of programming variables (*e.g*., duration, pre- and during-exercise feeding, accumulated fatigue, intensity, *etc.*) affect glucose demand, its release into the blood by the kidneys and liver, and its uptake into the muscle, the impact of various exercise protocols is frequently investigated.

Seven studies have reported on blood glucose concentrations following an acute bout of HIFT (Coco et al., 2019; Fogaça et al., 2020; Kliszczewicz et al., 2017; Perciavalle et al., 2016; Shaw et al., 2015; Tibana et al., 2016; Timón et al., 2019). Three observed no differences between resting and post-exercise glucose concentrations (Coco et al., 2019; Perciavalle et al., 2016; Shaw et al., 2015), while the remaining documented an elevated response (Fogaça et al., 2020; Kliszczewicz et al., 2017; Tibana et al., 2016; Timón et al., 2019). Though the limitations discussed in this review about different, un-equated workouts being featured across studies are ever present, the inconsistencies observed in glucose responses may have more to do with sample characteristics. Pre- to post-exercise glucose concentrations were reported as being steady when the study involved a heterogenous sample (*i.e*., variable physical activity and training backgrounds) (Coco et al., 2019; Perciavalle et al., 2016; Shaw et al., 2015). These ranged from sedentary, novice, adults to professional athletes (*e.g*., CrossFit® athletes in possession of a “certificate of suitability for competitive sports” by a specialist in Sports Medicine, Competitive Bodybuilders). The primary concerns here are the contributions of standardized programming (*e.g*., absolute intensity loads) to relative workout intensity and familiarity with HIFT on workout efficiency. Because regular exercise improves glucose uptake and maintenance, *via* adaptations in physiological systems, a blunted response is to be expected in trained individuals compared to those who are typically sedentary (Kristiansen et al., 2000). Meanwhile, a more experienced trainee might be assumed to have had more opportunities to familiarize themselves with various movement pattern combinations and develop more effective and efficient pacing strategies. Unfortunately, only one study has investigated glucose responses to HIFT in sedentary, untrained adults (Shaw et al., 2015), and as previously discussed (see prior sections), the reported indicators of HIFT workout intensity (*i.e*., peak blood lactate was 5.95 millimoles per liter; average heart rate was ~53.5% of HRmax) were much less than those reported by other studies. The reduced intensity would have necessarily affected metabolic/glucose demands (Brooks et al., 2005). Likewise, only one pilot study (Mangine et al., 2021b) and less than a handful of abstract presentations (Kliszczewicz et al., 2021; Mangine et al., 2021a; Zeitz et al., 2021; Dexheimer et al., 2021) have begun investigating aspects about pacing strategy. Thus, the impact of training history and HIFT experience on glucose control remains largely unexplored and leaves much to be assumed when organizing study findings.

Nevertheless, changes in blood glucose have been noted in studies that have used more homogenous samples. Fogaça et al. (2020) reported elevated blood glucose concentrations following full HIFT training sessions (*i.e*., warm-up, a strength component, gymnastic component, and metabolic conditioning session) after participants consumed 6 mg/kg body mass of caffeine (+3.2 mmol/L) or placebo (+1.5 mmol/L), with the caffeine condition producing higher concentrations. In an earlier study, Tibana et al. (2016) reported elevated blood glucose following each full training session on two consecutive days. Although both days featured similar programming (*i.e*., the same relative intensity and volume schemes for Olympic lifting, a gymnastic skill-based component, and “workout-of-the-day” duration), the second day’s glucose response was significantly less. This might be explained by different workloads being completed and second day effort, but neither were reported. Likewise, even though participants were asked to avoid caffeine and maintain their normal dietary habits on each day, this was not verified, leaving the reasons behind the second day’s reduced blood glucose response unclear.

Elevated blood glucose has also been observed when individual workouts were assessed. Kliszczewicz et al. (2017) reported statistically similar elevations following “Grace” (+45.7%) and a 15-min AMRAP (+65.8%), and these coincided with similar lactate, heart rate, and insulin responses. Further, blood glucose returned to baseline at 1-h post-exercise while insulin concentrations were lower than baseline assessments. Participants completed both workouts fasted (4 h), 1 week apart at the same time of day. In a later study, Timón et al. (2019) also had participants complete two different HIFT workouts 72 h apart from each other, and reported elevated glucose concentrations. However, it is unclear when concentrations returned to baseline, as the next time point did not occur until 24-h post-exercise. Interestingly, glucose elevations on the second workout (+71.5%) exceeded those of the first (37.4%). The reasons for why this latter study observed statistical differences in glucose responses are currently unclear but can likely be attributed to several methodological differences existing between studies. For this specific measure, both studies carried a similar sample size (10 *vs*. 12 participants) but different definitions for being considered HIFT-trained (3 *vs*. 12 months), different durations between experimental visits (3 *vs*. 7 days), different fasted durations (4 *vs*. 8 h), and utilized different HIFT workouts. Any of these may have been responsible for the lack of consistency between these studies. While the exact details about each workout’s programming, aside from “Grace” (30 clean and jerks at 64.1 kg), may be found in Table 2, two notable differences were that Timón et al. (2019) programmed relative loads for power cleans in the second workout (40% 1RM equaling ~37.3 kg) and the difference in resultant pacing (workout 1 = 0.30 repetitions per second; workout 2 = 0.07 repetitions per second) was much greater than what occurred in the study by Kliszczewicz et al. (2017) (“Grace” = 0.15 repetitions per second; 15-min AMRAP = 0.30 repetitions per second). Though relative intensity of loads used during “Grace” and the 15-min AMRAP remain unknown, the combination of wall ball shots with relative power clean loads led to a much slower pace and likely a greater metabolic demand. It is advisable for future studies to consider relative intensity when explaining and discussing the context of physiological findings in relation to HIFT.

### Acute power output outcomes

An individual’s expression of power may represent a desired training outcome, or it could be used as a metric of readiness (*i.e*., how they are responding to and recovering from training). Unless it is stated otherwise (*e.g*., “for quality”), HIFT workouts consistently direct trainees to give maximal effort (*i.e*., AMRAP, TTC), which of course, is still auto-regulated. This directive is advantageous because it allows performance to be quantified by the individual’s score in a workout. It also allows progress to be monitored *via* performance changes in standardized workouts (*e.g*., “Cindy”, “Fran”, *etc.*) (Feito et al., 2018b; CrossFit, 2002). For example, progress is easily tracked by the changes in the number of repetitions completed during a standardized AMRAP (*e.g*., “Cindy”) or the TTC for standard workload (*e.g*., “Fran”). Improving one’s score in either of these workout types also means that more work was completed in the same amount of time (*i.e*., that the expression of power improved). However, it would be inconsistent with HIFT’s definition to regularly assign the same benchmark workouts (Feito et al., 2018b; CrossFit, 2019). Instead, indirect measures (*e.g*., vertical jump (VJ)) may be more consistently used to estimate an individual’s response to training and monitor fatigue. That is, once expected changes in power following HIFT workouts and or competition are better understood. Of the 47 articles reviewed, changes in acute power expression were an outcome measure in only five studies (Escobar, Morales & Vandusseldorp, 2017; Tibana et al., 2016, 2019b; Mate-Munoz et al., 2017, 2018).

One of the earliest studies to contribute evidence related to recovery was primarily designed to examine the consistency of metabolic parameters measured during the hero workout “Rahoi” (Escobar, Morales & Vandusseldorp, 2017). Though examining recovery was not a specific study aim, short-term recovery was revealed as a consequence of the investigation’s use of a familiarization session for a standardized HIFT workout and lack of intervention between trials. Briefly, participants completed a 12-min AMRAP, rested completely for 3 days, and then repeated the workout (140.2 ± 25.9 repetitions); beating their initial score (131.2 ± 27.2 repetitions). The improved repetition count suggests that 3 days of rest provided sufficient recovery and enabled a greater expression of power (*i.e*., more work completed in 12 min) when the workout was repeated. That said, an alternate conclusion that still implies sufficient recovery states that the first trial familiarized the participants and allowed them to formulate a more appropriate strategy for the second trial. It is not clear whether the participants had ever completed “Rahoi” prior to the study, but the authors did ensure that participants had sufficient experience with HIFT (>1 year) and the specific exercises. Nevertheless, it would seem unlikely for performance to improve on the second trial, even with a better strategy, without sufficient recovery. In fact, it is plausible that 3 days was more than sufficient to allow for complete recovery. The participants reported training on at least three sessions per week, which would equate to less time between their typical training session days. The design of this study might actually represent a reduction in the training stimulus, due to participants having more time to recover, and limit the practical application of these particular findings.

In a pair of studies, Mate-Munoz et al. (2017, 2018) examined changes in VJ height and kinetics following three types of HIFT workouts in nearly identical samples of collegiate-aged men with no HIFT experience. All testing sessions for both studies followed the same order (“Cindy” → Double-Under “Tabata”-style → 1-RM Power Clean testing → Power Clean AMRAP) where sessions were separated by 1 week and participants refrained from physical activity for 48-h prior to each session. Consequently, both studies suffered from the same limitation that earlier testing sessions and a lack of control with physical activity (before 48-h pre-exercise) could have influenced performance on subsequent testing sessions. Still, each session’s effect on power was predominantly the same. Both reported reduced VJ performance quantified by height (−6.5% to −6.8%), relative average power (−4.2% to −4.4%), total average power (−3.9% to −4.0%), and take-off velocity (−2.7% to −12.2%) within 3 min of completing “Cindy”. Likewise, the same VJ measures declined in both studies by 3.1–7.6% after a 5-min AMRAP of power cleans using a load equal to ~40% 1-RM. However, only the first study (Mate-Munoz et al., 2017) observed reductions VJ performance (−1.2% to 3.6%) after a “Tabata”-style (*i.e*., 8, 20-s intervals with 10 s of rest) workout with double rope skips (a.k.a., double-unders), whereas only a greater reduction in take-off velocity was noted in the second study (Maté-Muñoz et al., 2018). While this exception may be simply attributed to the variability in training status that would be present among different samples of novice trainees, more research is clearly needed for confirmation.

Though helpful for providing starting points, the previously mentioned studies within this section do not address the effect of workouts occurring on consecutive days. This is a distinct possibility since CrossFit®, as an organization, publishes workouts daily on their website (CrossFit, 2001), and many affiliates are open 6–7 days per week, may incorporate these published workouts or write their own, and are not likely to actively limit members from attending daily. Currently, only two investigations out of Brazil have looked at the effect of consecutive workouts on power expression (Tibana et al., 2016, 2019b). The first study (Tibana et al., 2016) examined changes in VJ power (peak and average) across two consecutive workout days that each prescribed a strength, gymnastic, and metabolic conditioning component but failed to adequately describe the extent of the participants’ HIFT experience. All that was known was that participants possessed at least 6 months of HIFT experience and an unknown amount of resistance training experience prior to enrollment. The importance of their status is relevant to their ability to recover from training. The results showed that each workout elicited a significant reduction in average VJ power, but these returned to pre-exercise values within 24-h (*i.e*., suggesting sufficient recovery). However, peak VJ power steadily improved after each workout and between workouts so that by 24-h post the second training session, peak VJ power was significantly greater than what was observed prior to the first training session. This could imply a potentiating effect across workout sessions, but since peak power is representative of the power expressed during a single instant, it might simply be a spurious observation for a highly variable metric. In a later study from the same laboratory (Tibana et al., 2019b), VJ height was estimated (from flight time) in a more experienced sample (*i.e*., 28.9 months of HIFT experience) before and after they competed in a 3-day, three-person team HIFT competition. While the first study showed recovered or improved VJ power within 24 h (Tibana et al., 2016), VJ height did not recover until 48-h post-competition in the later study. It is possible that the more demanding nature of the competition or the additional day of training contributed the participants’ need for additional rest. However, the lack of sensitivity of VJ height compared to VJ kinetics may have also contributed to this difference (Mathieu et al., 2017). Regardless, both studies demonstrate that individuals with at least 6-months of HIFT experience may recover from 2–3 consecutive days of training within 24–48 h. Still, the infinite possibilities for HIFT programming limits the generalizability of these findings. A considerable amount of research surrounding a greater variety of HIFT workouts is needed before the recovery from such workouts can be understood well enough to begin forming recommendations.

## Conclusions

High-intensity functional training is a strategy that variably incorporates functional movements from weightlifting, gymnastics, and traditional cardiorespiratory exercise into daily workouts, which are intended to be performed at high-intensity, to improve general physical preparedness (Feito et al., 2018b). The intentional ambiguity and breadth of this definition leaves open an almost infinite number of possibilities for its interpretation in practice. Indeed, the composition and structure of each workout may be different on every day within the same week, month, year, or ever. Regardless of composition and structure, workouts are often accompanied by instructions meant to encourage effort put forth by trainees (*e.g*., repetitions completed within a set amount of time, time taken to complete prescribed tasks, frequency of rest breaks, *etc.*), but this is ultimately self-regulated by the individual, and thus, may lead to a wide range of acute physiological responses. Although exponential increases in popularity and research have been observed over the last two decades (*i.e*., since HIFT’s formal inception) (Thompson, 2021; Feito, Brown & Olmos, 2019), collective information on any given topic is still relatively limited compared to more traditional exercise strategies and sports. There is an extreme lack of consistency in the populations, variables measured, and workouts examined across studies examining acute responses to HIFT. Consequently, developing any kind of generalized conclusions *via* meta-analysis or systematic review on any given HIFT-related physiological response would be premature at this time. Instead, synthesizing the available information into a scoping review to report the current collection of findings and highlight areas in need of attention is more appropriate.

This scoping review observed clear discrepancies in the frequency of specific variables reported across existing studies. For instance, the number of studies reporting indicators of intensity (*i.e*., lactate and heart rate) is overwhelmingly higher than any other outcome variable. Although there may be several valid explanations for this (*e.g*., simplicity, availability of equipment, cost), the fact remains that acute responses to HIFT cannot be well understood without more studies taking a holistic approach. Severe deficiencies were noted in the quantity of research investigating various biochemical responses (*e.g*., individual hormone responses, inflammatory and damage markers) and energy demands. Regarding energy demands, greater attention should be placed on determining the best options for overcoming the several physical limitations related to its measurement. Current methodologies limit the types of exercises that may be incorporated into workouts under investigation, which is stark contrast to the spirit of the training strategy. This problem must be solved before HIFT-related energy demands can be adequately and comprehensively covered.

Perhaps the only generalized conclusion that can be made about HIFT at this time is that it lives up to its name. All but two investigations that reported indicators of intensity found a variety of HIFT workouts elicited a lactate or heart rate response that could be classified as vigorous. At best, though, these metrics provide a summary of the overall workout’s intensity. They do not quantify the contribution of individual workout components, and this is important for workouts that include weightlifting and gymnastic components. Since absolute loads are often prescribed in HIFT, knowing the relative intensity of those loads is essential for understanding acute physiological responses. Likewise, the trainee’s individual ability and efficiency in performing various gymnastic movements can drastically impact their allowable workout pace, and thus their physiological response. Currently, no universal method exists for quantifying the relative contribution of each component to intensity and work completed exists. Without such a metric, fair comparisons between different HIFT workouts or studies will be highly subjective.

Another important observation noted in this review is the variability in sample characteristics reported across studies. This may be partially addressed by improving descriptions of the relative intensity of studied HIFT workouts. More importantly, however, more consistency is needed in reporting training history and how training status is defined. Trained or experienced participants have been described as having 3 months to several years of HIFT experience. There are individual qualities, beyond one’s physiology, which are difficult to learn after only a few months of training (*e.g*., gymnastics skill, weightlifting technique, familiarity with various workout structures) but will still impact the trainee’s approach to a workout, as well as their physiological response. Before universal agreement on experience classifications is possible, future studies must become more consistent with the degree of detail reported about participants. From these, systematic reviews and meta-analyses may be performed to identify classification thresholds.

## Additional Information and Declarations

### Data Availability

The following information was supplied regarding data availability: This literature review did not require raw data.
